# The Landscape of Phenotypic and Transcriptional Responses to Ciprofloxacin in Acinetobacter baumannii: Acquired Resistance Alleles Modulate Drug-Induced SOS Response and Prophage Replication

**DOI:** 10.1128/mBio.01127-19

**Published:** 2019-06-11

**Authors:** Edward Geisinger, Germán Vargas-Cuebas, Nadav J. Mortman, Sapna Syal, Yunfei Dai, Elizabeth L. Wainwright, David Lazinski, Stephen Wood, Zeyu Zhu, Jon Anthony, Tim van Opijnen, Ralph R. Isberg

**Affiliations:** a Department of Molecular Biology and Microbiology, Tufts University School of Medicine, Boston, Massachusetts, USA; b Department of Biology, Boston College, Chestnut Hill, Massachusetts, USA; c Department of Biology, Northeastern University, Boston, Massachusetts, USA; University of Washington

**Keywords:** *Acinetobacter*, DNA gyrase, fitness, fluoroquinolone, prophage, antibiotic resistance, ciprofloxacin, topoisomerase IV

## Abstract

The emergence of fluoroquinolone resistance in nosocomial pathogens has restricted the clinical efficacy of this antibiotic class. In Acinetobacter baumannii, the majority of clinical isolates now show high-level resistance due to mutations in *gyrA* (DNA gyrase) and *parC* (topoisomerase IV [topo IV]). To investigate the molecular basis for fluoroquinolone resistance, an exhaustive mutation analysis was performed in both drug-sensitive and -resistant strains to identify loci that alter ciprofloxacin sensitivity. To this end, parallel fitness tests of over 60,000 unique insertion mutations were performed in strains with various alleles in genes encoding the drug targets. The spectra of mutations that altered drug sensitivity were found to be similar in the drug-sensitive and *gyrA parC* double-mutant backgrounds, having resistance alleles in both genes. In contrast, the introduction of a single *gyrA* resistance allele, resulting in preferential poisoning of topo IV by ciprofloxacin, led to extreme alterations in the insertion mutation fitness landscape. The distinguishing feature of preferential topo IV poisoning was enhanced induction of DNA synthesis in the region of two endogenous prophages, with DNA synthesis associated with excision and circularization of the phages. Induction of the selective DNA synthesis in the *gyrA* background was also linked to heightened prophage gene transcription and enhanced activation of the mutagenic SOS response relative to that observed in either the wild-type (WT) or *gyrA parC* double mutant. Therefore, the accumulation of mutations that result in the stepwise evolution of high ciprofloxacin resistance is tightly connected to modulation of the SOS response and endogenous prophage DNA synthesis.

## Abstract (precis)

Fluoroquinolones have been extremely successful antibiotics due to their ability to target multiple bacterial enzymes critical to DNA replication, the topoisomerases DNA gyrase and topo IV. Unfortunately, mutations lowering drug affinity for both enzymes are now widespread, rendering these drugs ineffective for many pathogens. To undermine this form of resistance, we examined how bacteria with target alterations differentially cope with fluoroquinolone exposures. We studied this problem in the nosocomial pathogen A. baumannii, which causes drug-resistant life-threatening infections. Employing genome-wide approaches, we uncovered numerous pathways that could be exploited to raise fluoroquinolone sensitivity independently of target alteration. Remarkably, fluoroquinolone targeting of topo IV in specific mutants caused dramatic hyperinduction of prophage replication and enhanced the mutagenic DNA damage response, but these responses were muted in strains with DNA gyrase as the primary target. This work demonstrates that resistance evolution via target modification can profoundly modulate the antibiotic stress response, revealing potential resistance-associated liabilities.

## INTRODUCTION

Acinetobacter baumannii is a frequent cause of multidrug-resistant infections in hospitals and has been labeled a pathogen of critical priority for new drug development (1). This pathogen class has rapidly evolved a broad array of drug resistance mechanisms, limiting the usefulness of many widely used antibiotics. A prime example is the fluoroquinolone class of antibiotics. These drugs are widely used to treat infections caused by a range of Gram-negative and Gram-positive bacteria, but they have been rendered obsolete against most A. baumannii isolates due to extremely high frequencies of resistance (2–4). An understanding of how A. baumannii and related bacteria withstand treatment with fluoroquinolone antibiotics has the potential to lead to strategies to reverse or bypass resistance.

Fluoroquinolones inhibit DNA replication in bacteria by targeting two enzymes essential for DNA synthesis, the type II topoisomerase DNA gyrase (*gyrAB* genes) and topoisomerase IV (topo IV; *parCE*). These enzymes modulate DNA topology to maintain negative DNA supercoiling (DNA gyrase) or decatenate newly replicated DNA (topo IV), and in so doing, they break and religate DNA. Binding of fluoroquinolones to these enzymes traps them in an intermediate state that is bound to cleaved DNA, resulting in double-strand DNA breaks, blocked replication fork progression, and, at high drug concentrations, cell death (5).

Acquired resistance to fluoroquinolones commonly arises through stepwise mutations that disrupt the ability of the drug to bind its preferred target enzymes. In Gram-negative bacteria, including A. baumannii, these mutations typically arise first in *gyrA*, which encodes the GyrA subunit of DNA gyrase, the more sensitive of the two enzyme targets (6). In the presence of resistant GyrA, the less-sensitive topo IV (encoded by *parC*) becomes the target and the site of second-step resistance mutations. In addition to target site alterations, acquisition of mutations that upregulate drug efflux pumps or accessory genes that allow drug modification enable bacteria to develop fluoroquinolone resistance (7). A large fraction of A. baumannii isolates harbor target site mutations in *gyrA* and *parC* (8, 9) and mutations causing overproduction of one or more resistance-nodulation-division (RND) class efflux systems that act on fluoroquinolone drugs (AdeABC, AdeFGH, and/or AdeIJK [10–12]).

Acquired resistance mechanisms generally act in combination with intrinsic resistance strategies in a cumulative manner to raise the amount of fluoroquinolone antibiotic required to block bacterial growth. Regulated production of native efflux pumps contributes to intrinsic fluoroquinolone resistance in many bacteria (13). Of the RND systems in A. baumannii, native levels of AdeIJK in wild-type (WT) strains lacking acquired mutations have been shown to provide intrinsic resistance to fluoroquinolones (12). Whether regulated production of other efflux systems provides intrinsic fluoroquinolone resistance is less clear.

Another major strategy for intrinsic fluoroquinolone resistance is the activation of DNA damage repair pathways (14). DNA lesions caused by fluoroquinolone intoxication are processed to single-stranded DNA and subsequently induce the SOS repair response, resulting in derepression of many genes involved in DNA recombination, repair, and mutagenesis (15). Knockout mutations in a variety of DNA repair genes result in increased fluoroquinolone susceptibility in several species (14–24). In certain cases, the SOS response also induces mobile genetic elements that carry antibiotic resistance or toxin genes, potentially influencing the spread of resistance or virulence traits (25). The A. baumannii SOS repair response is noncanonical, lacking clear orthologs of many major players in other systems (26), and is characterized by a phenotypically variable response within cell populations (27). Inactivation of RecA, a central protein mediating DNA recombinational repair and SOS induction, or the RecBCD exonuclease V complex responsible for double-strand-break repair, greatly raises fluoroquinolone sensitivity in A. baumannii (28, 29). The role of the SOS response and other DNA repair systems, however, in the development of antibiotic resistance in this organism is largely unknown.

As part of a large-scale effort to characterize the molecular nature of intrinsic resistance of Acinetobacter baumannii to antimicrobials, we identified the entire spectrum of transposon insertion mutations that cause altered sensitivity to the fluoroquinolone antibiotic ciprofloxacin during growth in bacteriological culture. A number of studies have demonstrated that mutations that cause antibiotic hypersensitization in strain backgrounds lacking demonstrable resistance loci exhibit these effects independently of whether there are target site resistance mutations or antibiotic-inactivating enzymes present in the strains being interrogated (20, 30). We wanted to test this model by first identifying, via transposon sequencing (Tn-seq), loci that confer intrinsic fluoroquinolone resistance in a strain background having intact drug targets and then comparing them with intrinsic resistance loci identified in strains having drug target mutations in *gyrA* and *parC*. This analysis led to the surprising discovery that the levels of SOS induction and endogenous prophage activation by fluoroquinolones show dramatic dependence on the drug target that is preferentially poisoned.

## RESULTS

### Identification of Acinetobacter baumannii loci that confer altered sensitivity to ciprofloxacin.

**FIG 1:**
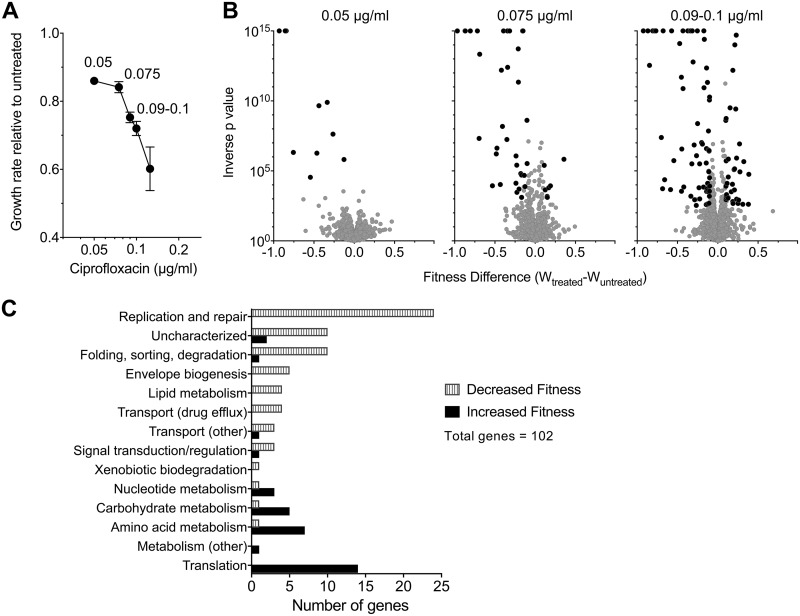
Tn-seq quantification of genome-wide mutant fitness in *A. baumannii* (*gyrA*^WT^ and *parC*^WT^) during growth with sub-MIC ciprofloxacin. (A) Graded concentrations of ciprofloxacin cause increasing degrees of growth inhibition. Transposon mutant libraries constructed in a strain background harboring WT alleles of the *gyrA* and *parC* genes were grown with ciprofloxacin at the concentrations indicated. Growth rate relative to untreated control was determined from bacterial density measurements. Data points show average ± SD (*n* ≥ 2). (B) Tn-seq fitness profiles during ciprofloxacin challenge. Mutant pools were grown with or without the indicated ciprofloxacin concentration, and the average fitness for each chromosomal gene was calculated. Change in fitness resulting from ciprofloxacin treatment relative to untreated controls is plotted against significance score resulting from parallel *t* tests. Data points shaded in black indicate gene knockouts causing significant alteration in fitness during drug challenge (magnitude of W_treated_ − W_untreated_, >0.1; FDR, <0.05). (C) Functional categories of significant gene hits determining fitness during challenge with 0.09 to 0.1 μg/ml ciprofloxacin. Information from KEGG and UniProt functional annotations and from orthologs in well-studied reference species were used to place genes into the listed broad categories.

To identify genes in which insertion mutations resulted in altered sensitivity to ciprofloxacin, ciprofloxacin concentrations below the MIC (Table 1) were identified that resulted in growth rates of A. baumannii ATCC 17978 in rich broth that were between 60 and 80% of that observed without antibiotics, as determined by optical density (OD) measurements (Fig. 1A). With increasing drug concentration, there is also a decrease in CFU, which the OD measurements underestimate (see Fig. S1B in the supplemental material). Three of these ciprofloxacin concentrations were chosen for further analysis (0.05, 0.075, and 0.09 to 0.10 μg/ml) to determine the relative fitness of insertion mutations when subjected to each of the antibiotic stress conditions. Multiple independent Tn*10* insertion pools (7 pools for 0.05 μg/ml and 11 pools for 0.075 and 0.09 to 0.10 μg/ml ciprofloxacin) having between 6,000 and 18,000 individual insertions (60,000 separate sites in all) were cultured in parallel in broth with or without ciprofloxacin for approximately 8 generations. DNA samples taken from the initial time point prior to growth (*t*_1_) and the final time point after 8 generations of growth (*t*_2_) were prepared from each of the pools. The insertion sites were then amplified preferentially and subjected to high-density sequencing, followed by determining the relative fitness of each insertion mutant based on the density of the reads (see Materials and Methods) (Data Set S1). Using accepted strategies, the fitness of each insertion mutant strain was calculated relative to the entire pool (31). To standardize the results across experiments, fitness values were normalized to insertions found in 18 neutral sites located in pseudogenes or endogenous transposon-related genes throughout the genome (“neutral” mutants), to allow an accurate quantitation of the representation of mutants relative to control insertions predicted to have no effect on growth (32). The normalized data from the individual insertion mutations were aggregated for each gene to calculate a mean fitness level for the entire spectrum of mutations found within a particular gene. The complete data sets were then displayed on an individual gene level as the growth rate changes for mutations relative to the growth rate of the entire pool (Fig. 1B). Candidate mutants were identified that showed lower or higher fitness levels based on the criteria that the false-discovery rate (FDR) *q* value was <0.05, the change in fitness (W_diff_) was >10%, and fitness value was derived from at least 3 independent insertion mutants (33) (see Materials and Methods). For this work, we refer to lesions resulting in lowered relative fitness during growth with ciprofloxacin as ciprofloxacin-hypersensitizing mutations, and we refer to the genes harboring these mutations as loci of hypersensitization.

**TABLE 1:** MICs of ciprofloxacin with WT and mutant *A. baumannii* strains

Strain	Amino acid change for:		Ciprofloxacin MIC (μg/ml)
	GyrA	ParC	
ATCC 17978 (WT)			0.25
EGA336	S81L		2–4
GVA41		S84L	0.25
GVA37	S81L	S84L	32

10.1128/mBio.01127-19.1 FIG S1 Features of transposon delivery plasmid pDL1073 and determination of growth rate changes as a function of ciprofloxacin concentrations. (A) pDL1073 features map. (B). Growth rate was determined by OD measurements (left) and CFU plate count measurements (right) with *A. baumannii* cultures grown without or with the indicated concentration of ciprofloxacin. Data points show average growth rate relative to untreated cultures ± SD (*n* ≥ 2). Growth rate measurement via CFU count was not determined for 0.125 μg/ml ciprofloxacin. Download FIG S1, PDF file, 0.2 MB. Copyright © 2019 Geisinger et al. 2019 Geisinger et al. This content is distributed under the terms of the Creative Commons Attribution 4.0 International license.

10.1128/mBio.01127-19.7 DATA SET S1 Tn-seq fitness data for WT. Download Data Set S1, XLSX file, 2.1 MB. Copyright © 2019 Geisinger et al. 2019 Geisinger et al. This content is distributed under the terms of the Creative Commons Attribution 4.0 International license.

Small increases in the dose of ciprofloxacin greatly increased the spectrum of ciprofloxacin hypersensitivity loci (Fig. 1B, black). At a dose causing approximately 15% growth inhibition, mutations in only 10 genes passed the criteria for lower fitness relative to the rest of the pool in the presence of the drug. These included insertions in two genes involved in double-strand-break repair (*recB* and *ruvA*, encoding subunits of exonuclease V and the Holliday junction helicase); the major egress pump which is often found overproduced in clinical strains having high-level fluoroquinolone resistance (*adeIJK*); and *ctpA*, a periplasmic protease shown to be a target of mutations that augment β-lactam resistance in strains lacking the *bfmRS* global regulatory system (34) (Data Set S1). Increasing the drug dose had two effects on expanding the spectrum of hypersensitivity loci. First, although the number of hypersensitivity loci that contribute to the enzymology of DNA repair increased from 2 members to 20 in the high-dose regimen, this expansion largely involved hitting additional subunits of the same complexes or backup systems of the enzymes identified in the low-dose regimen (*recBCD, sbcCD*, and *ruvABC*) (Data Set S1). This emphasizes the importance of protecting against double-strand breaks caused by fluoroquinolone-poisoned DNA gyrase (35). Second, increasing the dose resulted in hypersensitivity loci in cell envelope integrity proteins, additional protein-processing enzymes, and a MATE class proton-driven efflux pump (*abeM*) shown to export ciprofloxacin and other antibiotic compounds when cloned in Escherichia coli (36) (Data Set S1 and Fig. 1C). Interestingly, increasing the drug dose did not implicate the two other major RND efflux systems in protecting from ciprofloxacin stress even though they are known to provide low-level resistance after overproduction (12). This may be explained by the fact that the *adeIJK* system is the only RND egress pump known to have a high basal level of expression in WT strains lacking acquired resistance mutations, while the inducing signals for the other systems have not been identified (12). Strikingly, at the higher drug doses, mutations in *adeN*, which encodes the negative regulator of *adeIJK,* increased the fitness of A. baumannii relative to the rest of the pool (Data Set S1). These data argue strongly that the primary efflux pumps involved in intrinsic protection from fluoroquinolone stress are AdeIJK and AbeM.

In addition to hypersensitivity loci, mutations were identified at the highest drug dose that resulted in increased fitness relative to the insertion pool (Fig. 1B and C and Data Set S1). The mutations that most frequently increased fitness targeted nonessential components of the protein translation machinery, particularly enzymes that posttranslationally modify tRNA, rRNA, and assembly of ribosomal protein complexes. That disruption of this circuit is being associated with increased drug resistance is consistent with studies showing that a spectrum of antibiotic-resistant isolates in different species evolve mutations that cause a lower translation rate (37, 38). The results are also consistent with a study demonstrating that lowering ribosomal synthesis increases resistance to ciprofloxacin by restoring an optimal balance between protein and DNA synthesis levels during DNA stress (39). Most notable among the insertions identified were those in *gidA*, which is part of a complex involved in 5-methylaminomethyl-2-thiouridine (mnm^5^s^2^U34) modification of tRNAs (Data Set S1) (40). We have previously identified this gene as an additional target of mutations bypassing drug hypersensitivity resulting from a loss of *bfmRS* (34), indicating the tight connection between mutations in this gene and drug resistance.

### Deletion mutants have drug sensitivities predicted by Tn-seq.

**FIG 2:**
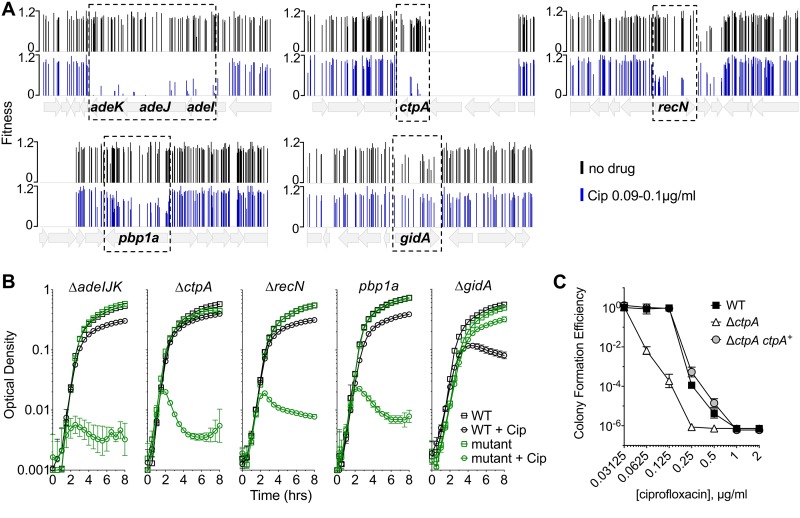
Magnitude of growth impairment is predicted by the severity of the Tn-seq fitness defect. (A) Tn-seq fitness profiles of transposon mutant pools constructed in a *gyrA*^WT^ *parC*^WT^ mutant strain. Pools were grown without or with ciprofloxacin (Cip) at 0.09 to 0.1 μg/ml. Bars show fitness values of each transposon mutant at the indicated locus across all tested pools. (B) Growth of pure cultures of WT or the indicated mutant in the absence or presence of ciprofloxacin (0.09 μg/ml for Δ*ctpA* and *pbp1A* mutants, 0.1 μg/ml for Δ*adeIJK* and *ΔrecN* mutants, and 0.15 μg/ml for Δ*gidA* mutant). The *pbp1a* mutant tested was *pbp1a*(N178TfsX27). Data points show geometric mean ± SD (*n* = 3). (C) Δ*ctpA* mutant shows lowered efficiency of colony formation on ciprofloxacin medium. WT bacteria with control plasmid (pEGE244), Δ*ctpA* mutant with control plasmid (pEGE244), and Δ*ctpA* mutant with plasmid-borne *ctpA*(pGVE41) were grown on increasing concentrations of ciprofloxacin on solid LB agar medium. Data points represent the geometric mean ± SD (*n* = 3).

Targeted deletion mutations were constructed in nonessential genes predicted to have altered drug sensitivity in the presence of ciprofloxacin. The mutations were chosen based on their fitness in the Tn-seq analysis, the magnitude of the effects predicted, and differing functional categories (Fig. 1C). For instance, mutations in the egress pump-encoding *adeIJK* showed extremely poor fitness and were rarely recovered after growth in ciprofloxacin (Fig. 2A). Similarly, the insertions in *ctpA* showed very low fitness. In contrast, although ciprofloxacin treatment lowered fitness for mutants lacking the penicillin binding protein 1A (PBP1A), these mutations clearly had weaker effects in the Tn-seq analysis. When this set of targeted mutants was analyzed further, the loss of *adeIJK, recN,* and *ctpA* all resulted in heightened drug sensitivity, as measured by growth in broth with or without ciprofloxacin (Fig. 2B). A *pbp1A* knockout strain harboring a frameshift mutation (see Materials and Methods) also showed heightened drug sensitivity (Fig. 2B). In addition, we assessed the ability of the *ctpA* deletion strain to form colonies on solid medium containing graded levels of ciprofloxacin. At drug concentrations that were below the MIC for the WT, the *ctpA* deletion mutation resulted in lower colony formation efficiency, and this hypersensitivity phenotype could be rescued by the reintroduction of *ctpA* on a plasmid (Fig. 2C). In contrast to these hypersensitivity loci, the deletion of *gidA-*encoded tRNA modification enzyme resulted in enhanced fitness in the presence of ciprofloxacin, with increased yields in broth cultures exposed to 0.15 μg/ml of antibiotic (Fig. 2B).

### Identification of loci that result in altered ciprofloxacin sensitivity in A. baumannii target site mutants.

**FIG 3:**
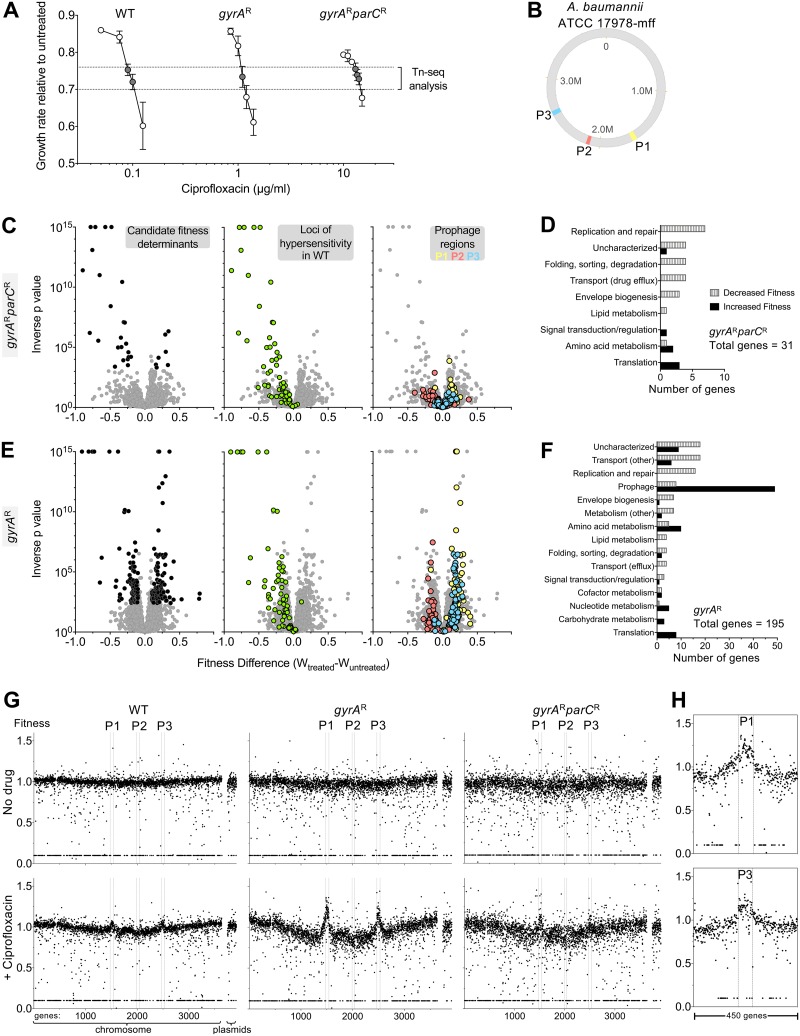
Acquisition of *gyrA* resistance allele dramatically alters *A. baumannii* Tn-seq profile during ciprofloxacin challenge. (A) Transposon pools constructed in strains harboring resistance alleles in *gyrA* or both *gyrA* and *parC* require increasing concentrations of ciprofloxacin to result in growth inhibition. Growth rate inhibition relative to untreated pools based on bacterial density measurements was plotted as in Fig. 1A. *gyrA*^WT^ *parC*^WT^ mutant growth data are from an identical experiment shown in Fig. 1A and are displayed to allow comparison to behavior of drug resistant mutants. Data points show average ± SD (*n* ≥ 2). Samples from cultures with 25 to 30% growth inhibition (dotted lines) were processed for Tn-seq. (B) Location of prophage regions (P1 to P3) on *A. baumannii* 17978-mff chromosome map. Prophage positions were identified by using the PHASTER database (68). (C to F) *gyrA* resistance allele influences Tn-seq fitness profiles associated with ciprofloxacin stress. Mutant pools were challenged with drug concentrations that resulted in equivalent 25 to 30% growth inhibition (*gyrA*^r^ mutant, 1.1 μg/ml; *gyrA*^r^ *parC*^r^ mutant, 13 to 14 μg/ml). (C and E) Tn-seq fitness scores for each chromosomal gene with the indicated strain were calculated and visualized as in Fig. 1B (left). Middle and right graphs show the identical data set, with highlighting of loci for which knockout causes ciprofloxacin hypersensitization in the WT genetic background (green), or loci within prophages (color indicated in key). (D and F) Gene hits associated with significant changes in fitness during treatment were placed into functional categories as in Fig. 1C. (F) Tn-seq hits resulting from *gyrA*^r^ mutant libraries treated with ciprofloxacin are enriched in prophage genes. (G) Tn-seq fitness scores resulting from ciprofloxacin challenge show genome positional bias that is greatly amplified in *gyrA*^r^ mutant pools. Average per-gene Tn-seq fitness values are plotted in order of gene position on the chromosome or on plasmids pAB1 to pAB3. Boundaries of prophage regions (P1 to P3) are indicated by vertical dotted lines. Top, no-drug control. Bottom, ciprofloxacin was added at the concentrations indicated in panel A (WT, 0.09 to 0.1 μg/ml; *gyrA*^r^ mutant, 1.1 μg/ml; *gyrA*^r^ *parC*^r^ mutant, 13 to 14 μg/ml). (H) Expanded view of per-gene Tn-seq fitness scores in regions surrounding prophages P1 and P3 for *gyrA*^r^ mutant treated with 1.1 μg/ml ciprofloxacin.

Most of the current clinical isolates of A. baumannii are resistant to fluoroquinolones, and these isolates commonly have the *gyrA*(S81L) and *parC*(S84L) target site mutations that lower the affinity for these antibiotics (41). To determine the spectrum of insertions that cause altered sensitivity to ciprofloxacin in strains having resistance alleles, *gyrA*(S81L) (here referred to as *gyrA*^r^) and *gyrA*(S81L) *parC*(S84L) (referred to here as *gyrA*^r^ *parC*^r^) mutants were generated, and each strain was subjected to Tn*10* mutagenesis. Pools totaling more than 70,000 insertion mutations were constructed in each background. Insertion pools were challenged with ciprofloxacin, using drug concentrations below the MIC (Table 1) that resulted in 25 to 30% growth inhibition for each strain (1.1 μg/ml for the *gyrA*^r^ mutant, 13 to 14 μg/ml for the *gyrA*^r^ *parC*^r^ double mutant; Fig. 3A). The spectrum of insertions that resulted in hypersensitivity to ciprofloxacin in the *gyrA*^r^ *parC*^r^ double-mutant strain backgrounds was very similar to that for the WT (Fig. 3C and D). In fact, almost every ciprofloxacin-hypersensitive locus in the double-mutant background was identified previously in the WT (Fig. 3C, green circles, and Data Sets S1 and S3). In addition, there was a number of hypersensitivity loci identified in the WT pools that did not pass the discovery criteria in the double mutant (FDR < 0.05; W_diff_ > 0.1). A number of these below-threshold candidates in the *gyrA*^r^ *parC*^r^ double-mutant strain background encoded subunits of the proteins identified as ciprofloxacin-hypersensitive loci (Fig. 3C, green circles, and Data Set S3). These results are similar to what we had observed in our graded series of drug treatments of insertion pools in the WT strain, indicating that the results from the WT strain and the drug-resistant double mutant are largely the same.

The results from the *gyrA*^r^ single-mutant background, however, diverged greatly from those with the WT and the *gyrA*^r^ *parC*^r^ double mutant (Fig. 3E). A large fraction of insertions were identified that altered drug sensitivity to ciprofloxacin, with a surprising number showing increased fitness during drug exposure (Fig. 3E). Over 40 of the insertions that exhibited increased fitness were located in putative prophage genes (Fig. 3E and F, blue and yellow circles) from two of the three predicted phages integrated into the bacterial chromosome (Fig. 3B and Data Set S2). No such fitness changes were seen in the WT (Fig. S2 and Data Set S1) or *gyrA*^r^ *parC*^r^ double mutant (Fig. 3C and Data Set S3). To analyze this result further, the normalized fitness of mutations in each gene was plotted as a function of position on the chromosome. In the absence of antibiotic, there was no clear positional effect of altered fitness levels along the length of the chromosome (Fig. 3G). In contrast, in the presence of antibiotic, there was an apparent increase in fitness levels centered within chromosomal locations harboring prophages P1 and P3 in the *gyrA*^r^ single mutant (Fig. 3G). Although some of this effect could be explained by a loss of prophage gene function resulting in enhanced fitness, insertions in chromosomal regions near, but outside, the prophage boundaries similarly showed apparent increases in fitness relative to the rest of the chromosomal insertions (Fig. 3G and H). As fitness levels are measured by counting the number of reads in specific regions of DNA, this phenomenon is consistent with selective local amplification of chromosomal material that initiates within these prophages, extending outward from the integration sites into nearby DNA regions. Deleting both P1 and P3 in the *gyrA*^r^ background resulted in increased density during bacterial entry into postexponential phase in the presence of ciprofloxacin concentrations that approximate the MIC (Fig. S2B). This further supports the model that drug-induced amplification of prophage locus DNA was a major reason for heightened Tn-seq fitness values associated with transposons in these regions.

10.1128/mBio.01127-19.2 FIG S2 Relationship between prophages and Tn-seq fitness in *A. baumannii*. (A) Sub-MIC ciprofloxacin treatment of *A. baumannii* with *gyrA*^WT^ and *parC*^WT^ alleles does not significantly alter Tn-seq fitness values assigned to prophage region genes. The Tn-seq data set shown in Fig. 1B (WT background ± treatment with ciprofloxacin 0.09 to 0.1 μg/ml) was reanalyzed to highlight fitness values associated with genes within prophage regions P1 to P3. Prophage regions are highlighted with color indicated in the key. (B) Effect of prophages P1 and P3 double deletion on growth of *A. baumannii gyrA*^r^ mutant strain in presence and absence of ciprofloxacin. Growth of the deletion mutant compared to the P1^+^ P3^+^ isogenic *gyrA*^r^ mutant strain was measured in LB medium ± ciprofloxacin at the indicated concentration by monitoring the OD change in microtiter format, as described in Materials and Methods. Data points show geometric mean ± SD (*n* = 3). Download FIG S2, PDF file, 0.7 MB. Copyright © 2019 Geisinger et al. 2019 Geisinger et al. This content is distributed under the terms of the Creative Commons Attribution 4.0 International license.

10.1128/mBio.01127-19.8 DATA SET S2 Tn-seq fitness data for *gyrA*^r^ mutant. Download Data Set S2, XLSX file, 0.8 MB. Copyright © 2019 Geisinger et al. 2019 Geisinger et al. This content is distributed under the terms of the Creative Commons Attribution 4.0 International license.

10.1128/mBio.01127-19.9 DATA SET S3 Tn-seq fitness data for *gyrA*^r^ *parC*^r^ mutant. Download Data Set S3, XLSX file, 0.8 MB. Copyright © 2019 Geisinger et al. 2019 Geisinger et al. This content is distributed under the terms of the Creative Commons Attribution 4.0 International license.

### Two prophage regions are selectively amplified in response to ciprofloxacin in the *gyrA*^r^ single mutant.

**FIG 4:**
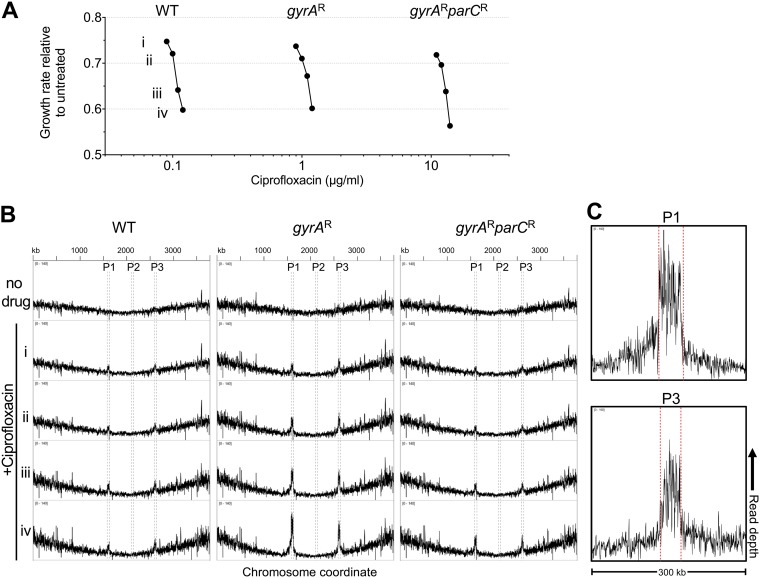
Ciprofloxacin-induced amplification of prophage DNA in strains harboring the *gyrA*^r^ single-step resistance genotype. (A) Pure cultures of strains of the indicated genotype were challenged with graded ciprofloxacin doses resulting in four levels of growth inhibition (Roman numerals). (B) DNA content from each culture was analyzed by deep sequencing. The *x* axis indicates nucleotide position along the *A. baumannii* ATCC 17978-mff chromosome. The *y* axis indicates normalized read depth (0 to 140 counts per million). Boundaries of prophage regions P1 to -3 are indicated by dashed lines. Roman numerals indicate the level of growth inhibition caused by ciprofloxacin. Data are representative of two independent experiments. (C) Expanded view of 300-kb window showing amplification of genomic regions surrounding prophages P1 and P3. The *y* axis indicates normalized sequencing read depth (0 to 160 counts per million).

**FIG 5:**
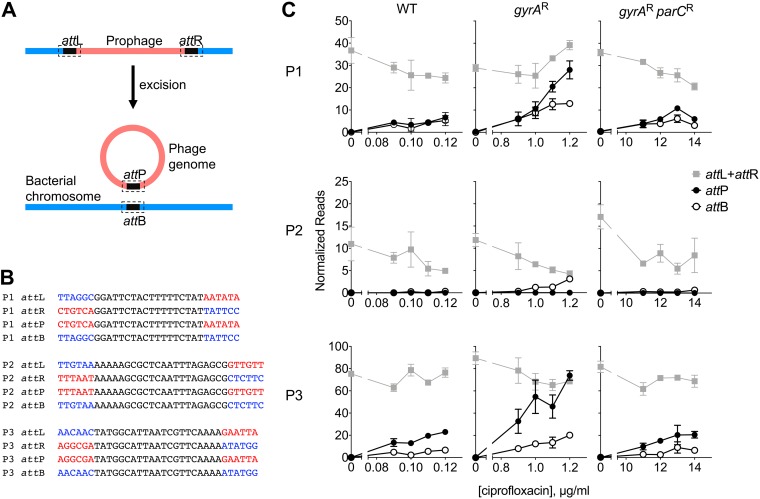
Enhanced frequency of prophage excision and circularization in *gyrA*^r^ single mutant. (A) Diagram of prophage excision at attachment sites (*att*; indicated by dashed boxes) showing generation of *attP* and *attB* sites after excision. (B) Prophage core *att* sequences (black) were determined using PHASTER database and BLAST analysis. Six-base-arm sequences flanking the core *att* sites were used to define *attL*, *attR*, *attP*, and *attB*. Prophage-specific DNA is shown in red, and chromosome-specific DNA is shown in blue. (C) Illumina whole-genome sequencing reads from experiment shown in Fig. 4 and an additional independent experiment were searched using the sequences shown in panel B. Read counts were normalized against total library size and displayed as counts per 10 million reads. Data points show average of normalized read count ± range (*n* = 2).

As an additional test of the model that there is induction of DNA synthesis in the region surrounding two of the chromosomally located prophage clusters, we directly analyzed the chromosomal DNA content in the WT and *gyrA*^r^ and *gyrA*^r^ *parC*^r^ mutant strains responding to drug treatment. Purified single colonies from each strain were grown in broth culture for 3.5 h in the presence of four different concentrations of ciprofloxacin that ranged from ∼25 to 50% growth inhibition and compared to bacteria grown in the absence of drug (Fig. 4A). DNA was then prepared from each of the cultures and subjected to whole-genome sequencing using an average read length of 100 bp. The density of these individual short reads was plotted as a function of the chromosomal coordinates to identify regions of chromosomal DNA that were selectively amplified in the presence of drug (Fig. 4B). Analysis of the *gyrA*^r^ single mutant showed hyperamplification of prophages P1 and P3, with read density in the prophage regions observed as a function of drug concentration. In contrast, there was little evidence of this selective amplification in the WT strain, while the *gyrA*^r^ *parC*^r^ mutant largely reversed these effects. Consistent with the Tn-seq data, there was amplification of DNA extending beyond the prophage-chromosomal DNA junction, indicating that drug-driven DNA synthesis was initiated *in situ* and continued beyond the ends of the prophages into adjacent chromosomal DNA (Fig. 4C). In addition to this apparent *in situ* DNA amplification, P1 and P3 excision and phage genome circularization could be detected. To measure circularization and excision, the presence of *attB* and *attP* loci, which are absent in the intact prophage, was identified and quantified in the sequencing reads (Fig. 5A and B). Excision and circularization were detected at frequencies that increased with increasing drug dose in the *gyrA*^r^ strain (Fig. 5C). Strikingly, increasing drug concentrations also resulted in an excess of *attP* copies relative to *attB* (Fig. 5C), consistent with replication of circular phage genomes and excision from the chromosome. In contrast, the efficiency of such events was muted or close to background in either the WT or the *gyrA*^r^ *parC*^r^ backgrounds (Fig. 5C). We conclude that in a *gyrA*^r^ background, selective blockage of the *parC*-encoded topo IV protein resulted in DNA synthesis induction and phage genome multiplication in these two prophage regions.

**FIG 6:**
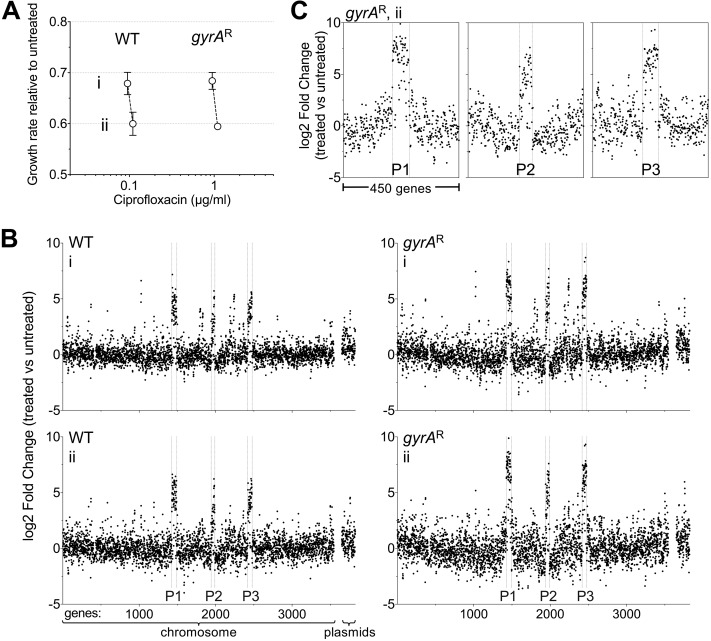
Ciprofloxacin challenge results in activation of prophage gene expression that is heightened in *gyrA*^r^ single mutants. (A) Strains of the indicated genotype were challenged with ciprofloxacin concentrations that resulted in two levels of growth inhibition relative to no treatment (i, ∼30% growth inhibition; ii, ∼40% growth inhibition). Data points show average ± SD (*n* = 3). (B). RNA-seq transcriptional profiles of cultures in panel A. Fold change (log_2_) of each gene (treated versus untreated) was plotted in order of position on the chromosome or plasmids (pAB1 to pAB3). rRNA and tRNA genes were excluded from RNA-seq analysis, resulting in different gene number assignments from those in Fig. 3G. Boundaries of prophage regions P1 to P3 are denoted by vertical dotted lines. Roman numerals indicate growth inhibition level. (C) Expanded views of RNA-seq log_2_ fold change ratios for genes surrounding P1 to P3 in *gyrA*^r^ single mutant (condition *ii*, 1.1 μg/ml ciprofloxacin).

To determine if the transcription of prophage genes is specifically amplified in the *gyrA*^r^ mutant relative to the WT, the two strains were grown in triplicate cultures in two concentrations of antibiotic for 3.5 h that gave between 30 and 40% growth inhibition over approximately 7 generations (Fig. 6A). The cells were then extracted and subjected to RNAtag-seq analysis (42), and the ratio of transcription for each gene in the presence/absence of ciprofloxacin was displayed as a function of chromosomal map position (Fig. 6B and Data Set S4). There was preferential amplification of transcription of prophage genes in the presence of antibiotic treatment in both strain backgrounds (Fig. 6B). Furthermore, transcription was hyperactivated in all three prophages, including prophage 2, which showed no evidence of preferential DNA amplification (compare Fig. 4B and 6B). A high proportion of genes in all three prophages showed transcriptional responses to ciprofloxacin that were significantly higher in the *gyrA*^r^ single mutant than in the WT (Fig. 6B and S3). In *gyrA*^r^ single mutants, enhanced expression in response to ciprofloxacin extended beyond the prophage-chromosomal DNA junctions with prophage 1 and, to some extent, with prophage 3 (Fig. 6C), consistent with increased DNA template availability partially contributing to heightened transcription in this strain background. Hyperexpression in response to ciprofloxacin terminated at the prophage-chromosomal DNA junctions with prophage 2, consistent with the observation that this region experienced no DNA amplification (Fig. 6C). These results indicate that preferential blockage of topo IV in the single mutant results in hyperactivation of prophage transcripts.

10.1128/mBio.01127-19.3 FIG S3 Comparison of transcriptional induction of prophage genes in WT and *gyrA*^r^ mutant strains in response to ciprofloxacin reveals enhanced induction in the *gyrA*^r^ single mutant. Bars show log_2_ fold change ± SEM (*n* = 3) in RNA-seq transcription level comparing ciprofloxacin-treated versus untreated control for either WT (black) or *gyrA*^r^ single mutant (blue). For each strain, the ciprofloxacin dose caused an equivalent 40% inhibition of growth (condition *ii* from Fig. 6). In unpaired *t* tests, 58/67 P1 genes, 24/51 P2 genes, and 45/60 P3 genes had a *P* value of <0.05 (Data Set S4). Download FIG S3, PDF file, 0.07 MB. Copyright © 2019 Geisinger et al. 2019 Geisinger et al. This content is distributed under the terms of the Creative Commons Attribution 4.0 International license.

10.1128/mBio.01127-19.10 DATA SET S4 RNA-seq data. Download Data Set S4, XLSX file, 2.4 MB. Copyright © 2019 Geisinger et al. 2019 Geisinger et al. This content is distributed under the terms of the Creative Commons Attribution 4.0 International license.

### Ciprofloxacin-induced SOS response is selectively enhanced in the *gyrA*^r^ single mutant.

**FIG 7:**
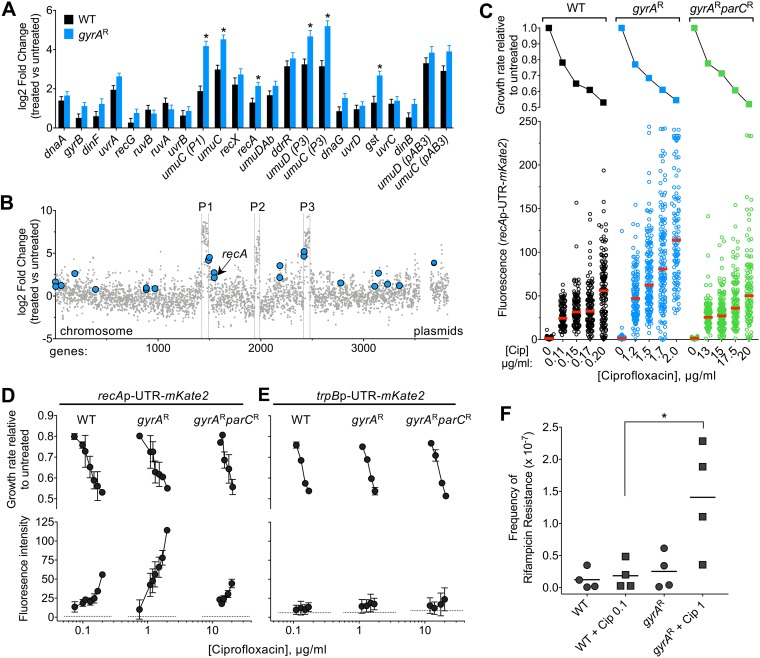
Enhanced SOS response induction in *gyrA*^r^ single mutants exposed to sub-MIC ciprofloxacin. (A and B) SOS response genes are induced during growth with ciprofloxacin. (A) RNA-seq data reveal DNA damage/SOS response induction during growth with ciprofloxacin. Bars show log_2_ fold change ± SEM (*n* = 3) for WT (black) or *gyrA*^r^ single mutant (blue) treated with ciprofloxacin concentrations resulting in ∼40% growth inhibition (condition *ii* from Fig. 6). *, *P* < 0.05, unpaired *t* test. (B) Location of DNA damage/SOS response genes induced in *gyrA*^r^ single-mutant strain. *x* axis indicates gene position along the *A. baumannii* chromosome, and *y* axis indicates the log_2_ fold change (1.1 μg/ml Cip versus untreated, *gyrA*^r^ single mutant) from previously presented RNA-seq data. (C) Population fluorescence analysis of *recA* reporter induction from a representative experiment. Strains of the indicated genotype harboring pCC1(*recA-mKate2*) were cultured with or without ciprofloxacin as in RNA-seq experiments. Top, growth inhibition relative to untreated control was calculated. Bottom, mKate2 intensity per cell was measured by fluorescence microscopy; each data point represents mean mKate2 intensity of a single cell, and bars indicate median values. (D and E) Average inhibition values (top) or average of median fluorescence values (bottom) from multiple independent experiments examining fluorescence reporter levels as in panel C are displayed. Strains of the indicated genotype harbored *recA-mKate2* (D) or pCC7 (*trpB* promoter fused to *mKate2* (E). Data points represent mean ± SD at each concentration (*n* ≥ 2). Dotted lines denote average fluorescence intensity of untreated samples. (F) Rifampin resistance mutation frequency in WT or *gyrA*^r^ mutant strains grown in the absence or presence of the indicated ciprofloxacin dose for 3 h. Data points show the efficiency of colony formation on LB agar plates containing rifampin compared to LB agar without antibiotic from *n* = 4 independent experiments. *, *P* = 0.032, unpaired *t* test.

Intoxication of bacterial topoisomerase enzymes by fluoroquinolone antibiotics induces DNA damage, driving an SOS response (16, 43). We investigated the extent to which ciprofloxacin-induced hyperactivation of prophage gene expression coincided with SOS response induction and whether the *gyrA* and *parC* resistance alleles influenced this response. Several genes associated with the SOS response (27, 43, 44) showed heightened transcription as a consequence of ciprofloxacin treatment (Fig. 7A). For several SOS genes, induction of transcripts was significantly higher in the *gyrA*^r^ mutant than in the WT (Fig. 7A, asterisks). These included genes adjacent to prophage-chromosomal DNA junctions (*umuC* and *umuD* paralogs) as well as those not linked to prophages (*recA* and *gst*; Fig. 7B). RecA is a key component of the SOS response that is induced by DNA damage in A. baumannii and is critical for withstanding ciprofloxacin stress independent of the background resistance genotype (see Data Sets S1 to S3). To analyze the interplay of SOS induction by ciprofloxacin with target availability at the level of single cells, we utilized a plasmid-based transcriptional fusion of the *recA* regulatory elements (promoter and 5′-untranslated region [5′-UTR]) to the fluorescent reporter *mKate2* (45). WT, *gyrA*^r^ mutant, and *gyrA*^r^ *parC*^r^ mutant strains harboring the reporter fusion were cultured in the presence of graded levels of ciprofloxacin, and reporter signal was measured in individual cells by fluorescence microscopy (see Materials and Methods). Increasing sub-MIC doses of ciprofloxacin caused increasing degrees of induction of the *recA* reporter in all strain backgrounds (Fig. 7C and D). Notably, reporter activity was approximately 2-fold higher in the *gyrA*^r^ single mutant than in the WT or double mutant at equivalent levels of growth inhibition (Fig. 7C and D). Various degrees of *recA* induction within populations of *gyrA*^r^ single-mutant cells were observed, and this variability roughly matched that observed with WT (Fig. 7C and S4). Increased signal in the *gyrA*^r^ mutant strain was not observed with a control reporter fusion to a gene that is nonresponsive to ciprofloxacin (*trpB*p-UTR [45]) (Fig. 7D), indicating that the SOS transcriptional response was specifically enhanced as a consequence of ciprofloxacin inhibition of topo IV.

10.1128/mBio.01127-19.4 FIG S4 Fluorescence microscopy analysis of SOS response in individual cells subjected to ciprofloxacin. Strains harboring *recA-mKate2* were cultured without (top) or with the noted concentration of (bottom) ciprofloxacin and analyzed by fluorescence microscopy, as described in the legend to Fig. 7C. Phase-contrast (rows 1 and 3) and fluorescence (rows 2 and 4) microscopy images used to quantify *recA-mKate2* signal in Fig. 7C are shown. Download FIG S4, PDF file, 0.06 MB. Copyright © 2019 Geisinger et al. 2019 Geisinger et al. This content is distributed under the terms of the Creative Commons Attribution 4.0 International license.

We hypothesized that the enhanced ciprofloxacin-induced SOS response in the *gyrA*^r^ single-mutant strain leads to increased mutagenesis. To measure mutagenesis levels, we calculated the frequency of rifampin-resistant mutants in cultures of WT or *gyrA*^r^ mutant after 3 h of growth in the absence or presence of sub-MIC ciprofloxacin doses that result in 10 to 15% growth inhibition (see Materials and Methods). The *gyrA*^r^ mutant bacteria exposed to ciprofloxacin showed a higher frequency of rifampin-resistant mutants than when strains were untreated or than the ciprofloxacin-treated WT (Fig. 7E). Together with the observation that *umuC* and *umuD* genes encoding low-fidelity DNA polymerase V show enhanced induction in *gyrA*^r^ mutant cells compared to the WT or *gyrA*^r^ *parC*^r^ mutant strain (Fig. 7A), these findings are consistent with the model that the enhanced SOS response resulting from ciprofloxacin inhibition of topo IV is linked to a higher frequency of mutagenesis in the *gyrA*^r^ single mutant.

## DISCUSSION

In this study, we exploited the dual-target nature of fluoroquinolone antibiotics to uncover how resistance alleles acquired in target enzymes modulate the landscape of intrinsic resistance and transcriptional responses to this class of drug. Using Tn-seq, we performed comprehensive screens for determinants of altered sensitivity to the fluoroquinolone drug ciprofloxacin in isogenic A. baumannii strains in which the drug preferentially targets either DNA gyrase or topo IV. We found that the spectra of genes contributing to intrinsic resistance were similar in genetic backgrounds in which both enzymes were WT or in which both enzymes had lowered drug sensitivity due to well-known acquired point mutations. Intrinsic resistance determinants identified in both backgrounds included the AdeIJK and AbeM efflux pumps, multiple subunits of the DNA recombination and repair machinery, a periplasmic protease CtpA, the cell wall transpeptidase PBP1A, and several proteins of unknown function. In contrast, the interaction of ciprofloxacin with the *gyrA*^r^ single mutant in which topo IV is the sensitive target dramatically altered the profile of genes that influence relative Tn-seq fitness. This altered fitness profile in *gyrA*^r^ *parC*^+^ mutant bacteria was shown to directly reflect the amplification of DNA in the vicinity of two endogenous prophages due to preferential poisoning of topo IV by ciprofloxacin. This study highlights the potential for specific genotype-condition interactions to create unexpected chromosome positional bias in Tn-seq fitness analysis (46). Prophage transcripts and the SOS pathway were also hyperactivated as a consequence of topo IV poisoning by ciprofloxacin, likely facilitating the initiation of synthesis of prophage DNA in the *gyrA*^r^ mutant strain.

**FIG 8:**
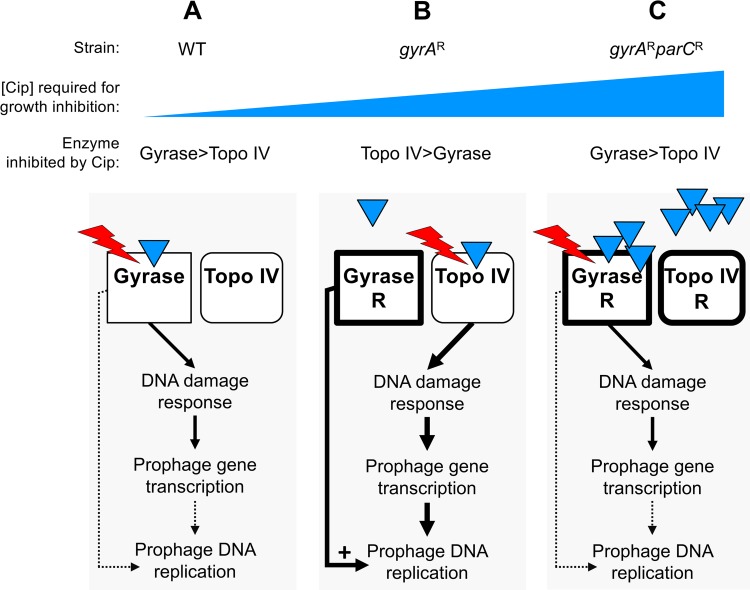
Model for resistance allele-dependent prophage amplification in *A. baumannii* exposed to sub-MIC fluoroquinolone stress. The model posits that prophage DNA replication depends on host DNA gyrase activity. (A) In WT cells, both gyrase (GyrA) and topo IV (ParC) are drug sensitive. Gyrase, which has higher affinity for ciprofloxacin (blue triangles), is effectively targeted by the drug. Ciprofloxacin-corrupted gyrase enzyme, indicated by red lightning symbol, results in double-strand DNA breaks that signal derepression of prophage gene expression. Prophage DNA replication cannot proceed efficiently, however, because gyrase function is blocked. (B) In *gyrA*^r^ single mutant cells, topo IV/ParC has higher affinity for ciprofloxacin than does the resistant gyrase and is the effective drug target. Topo IV corruption results in a robust DNA damage response and activation of prophage gene expression, and gyrase-dependent prophage replication (prophages 1 and 3) proceeds efficiently because GyrA is not drug inhibited. (C) In *gyrA*^r^ *parC*^r^ double-mutant cells growing at high drug concentrations, GyrA again has relatively higher affinity for ciprofloxacin than does ParC and becomes the effective target despite the S81L drug binding site alteration. The resulting DNA lesions induce the SOS response and prophage gene expression, but prophage replication does not proceed efficiently because gyrase function is again blocked.

Our data can be explained by the model shown in Fig. 8, if we assume that reactivation of A. baumannii prophages 1 and 3 requires the function of host DNA gyrase. In the WT and the *gyrA*^r^ *parC*^r^ double mutant, DNA gyrase is the effective target blocked by ciprofloxacin at growth-inhibitory sub-MIC drug concentrations. Prophage DNA synthesis is inefficient despite induction of the DNA damage response and prophage gene transcription because, as postulated by the model, efficient replication of the prophage genomes requires functional host gyrase (Fig. 8A and C). In contrast, in *gyrA*^r^ mutant bacteria, topo IV is the preferred target of intoxication by ciprofloxacin. This interaction causes DNA lesions that robustly stimulate the SOS pathway and prophage transcription; further, host DNA gyrase is available to facilitate efficient prophage genome replication because the GyrA(S81L) (*gyrA*^r^) variant is resistant to the intermediate concentrations of ciprofloxacin required for topo IV poisoning (Fig. 8B).

The central assumption of the model is plausible based on analogy with several other bacteriophage systems that have been shown to require host DNA gyrase for replication. Gyrase inhibitors (quinolones or aminocoumarins) inhibit phage DNA replication during lytic growth after infection (47–52) and disrupt induction of replicative transposition in Mu lysogens (53). Moreover, host gyrase is required for propagation of replication forks within supercoiled DNA substrates in reconstituted systems modeling phage lambda replication (54, 55). The importance of this enzyme class for replication of A. baumannii strain 17978 prophages is emphasized by the fact that they do not encode type II topoisomerases which are often encoded by bacteriophages to bypass a requirement for the host enzymes (56, 57).

An alternative model is that at the sub-MIC drug doses resulting in equivalent growth inhibition, gyrase poisoning results in DNA lesions that do not stimulate the SOS response above the threshold required for efficient prophage induction, in contrast to lesions caused by topo IV poisoning. Arguing against this model are the observations that transcription of the SOS response gene *recA* and genes from all three prophages are strongly induced (25- to 50-fold, Fig. 6B and 7C and D) above baseline in WT cells, and Tn-seq fitness results showing the relatively similar importance of DNA damage repair enzymes across all strain backgrounds (Data Sets S1 to S3). We showed that topo IV intoxication stimulated the SOS pathway to a greater extent than that caused by gyrase poisoning, potentially contributing to the robust activation of prophages. This is consistent with previous findings that topo IV and gyrase intoxication can be distinguished by several characteristics. Topo IV lesions result in slower inhibition of DNA synthesis and are thought to be more readily reversed by recombinational repair, resulting in lower cytotoxicity at given drug concentrations (16). These less toxic lesions could potentially expose more numerous or potent signals for the SOS response that could result in prophage induction.

In contrast to the induction of prophages 1 and 3, ciprofloxacin-induced DNA replication was not observed with prophage 2 despite activation of prophage gene transcription. One possible explanation is that prophage 2 is defective for DNA replication. We consider this unlikely because transposon insertions were unobtainable in a phage locus (ACX60_RS10145) encoding a putative Cro/Cl family repressor (Data Sets S1 to S3), indicating that this prophage has the potential for lytic replication in the absence of a protein controlling lysogeny maintenance. Consistent with the potential of all three prophages (including P2) for replication, mobilized DNA corresponding to each of the three prophages was detected in phage particles resulting from treatment of WT A. baumannii 17978 with mitomycin C, which damages DNA directly without dependence on interactions with DNA topoisomerases (44). An alternative explanation for the lack of prophage 2 DNA amplification observed with fluoroquinolone treatment in our study is that its replication depends on both DNA gyrase and topo IV.

The findings described here have implications for the evolution of antibiotic resistance in A. baumannii and potentially other organisms. They indicate that in the trajectory toward high-level fluoroquinolone resistance and intermediate states with moderate-level fluoroquinolone resistance (exemplified by the *gyrA*^r^ single mutant) are those that possess highest potential for prophage induction and mutagenesis during growth with continued drug exposure. This could influence how additional drug resistance alleles are acquired. Acquisition of the subsequent *parC* mutation resulting in high-level fluoroquinolone resistance would reset the proclivity for enhanced drug-induced SOS response and prophage amplification. It is notable that hyperamplified and hyperexpressed DNA in the *gyrA*^r^ single mutant includes multiple *umuC* and *umuD* paralogs encoding mutagenic DNA polymerases (Fig. 7A and B) (58), whose higher levels could potentially influence adaptation in bacterial populations during drug treatment. Moreover, these findings raise the possibility of enhanced horizontal transfer of phage-associated and phage-proximal genes as a consequence of fluoroquinolone-*gyrA*^r^ mutant interactions. Prophage clusters with sequence homology to P1 and P3 prophages exist in additional A. baumannii isolates, and in other isolates more divergent prophages can be found at identical chromosomal locations (59). Therefore, the presence of *gyrA*^r^ single mutant resistance may broadly modulate how mobile DNA and the A. baumannii chromosome respond to fluoroquinolone stress. The relationship between stepwise fluoroquinolone resistance and induction of the SOS response and prophages by this drug class may play out differently with Gram-positive organisms, in which topo IV is typically the sensitive initial target, as opposed to gyrase (6). If the dynamics of fluoroquinolone stress responses in such bacteria have features that accord with the model proposed here, WT strains with two sensitive *parC* and *gyrA* alleles may represent the state with higher potential for drug-induced prophage replication and SOS mutagenesis in Gram-positive organisms compared to derivatives that have acquired single-step target resistance mutations.

In summary, we have demonstrated that in the course of stepwise selection for high drug resistance, intermediate steps result in unexpected modulation of the antibiotic stress response leading to enhanced induction of prophages as well as the enzymatic machinery that drives the acquisition of drug resistance. Future work on analysis of proteins that modulate the survival of drug-resistant mutants should uncover strategies that allow these variants to be targeted therapeutically.

## MATERIALS AND METHODS

### Bacterial strains, growth conditions, and antibiotics.

The bacterial strains used in this work are described in Table S1. A. baumannii strains were derivatives of ATCC 17978. Bacterial cultures were grown at 37°C in lysogeny broth (LB; 10 g/liter tryptone, 5 g/liter yeast extract, 10 g/liter NaCl) in flasks with shaking or in tubes on a roller drum. Growth was monitored by measuring absorbance at 600 nm via a spectrophotometer. LB agar was supplemented with antibiotics (ampicillin [Amp], 50 to 100 μg/ml; carbenicillin [Cb], 50 to 100 μg/ml; kanamycin [Km], 10 to 20 μg/ml; amdinocillin; and ciprofloxacin) or sucrose as needed (Sigma-Aldrich).

10.1128/mBio.01127-19.5 TABLE S1 Bacterial strains and plasmids used in this study. Download Table S1, PDF file, 0.05 MB. Copyright © 2019 Geisinger et al. 2019 Geisinger et al. This content is distributed under the terms of the Creative Commons Attribution 4.0 International license.

### Molecular cloning and mutant construction.

The oligonucleotide primers and plasmids used in this study are listed in Table S2. *gyrA*(S81L) (*gyrA*^r^) and *parC*(S84L) (*parC*^r^) single point mutations were generated by cloning the respective genomic fragments in pUC18, followed by inverse PCR and self-ligation or amplification and substitution of a mutated gene fragment. In-frame deletions of *adeIJK*, *ctpA*, *recN*, and *gidA* were constructed as described previously (60). DNA fragments for deleting prophages P1 and P3 were cloned by PCR amplification of the corresponding *attB* locus and flanking regions from *gyrA*^r^ mutant strains exposed to ciprofloxacin. Constructs were subcloned in pSR47S and used to isolate A. baumannii mutants via homologous recombination with two selection steps (60). The *gyrA*^r^ *parC* ^r^ double mutant was isolated by selection of a derivative of the *gyrA*^r^ strain able to grow on LB agar containing 8 μg/ml ciprofloxacin. A *pbp1a* mutant (N178TfsX27) was isolated as a derivative of ATCC 17978 selected on LB agar containing 64 μg/ml amdinocillin.

10.1128/mBio.01127-19.6 TABLE S2 Oligonucleotide primers used in this study. Download Table S2, PDF file, 0.03 MB. Copyright © 2019 Geisinger et al. 2019 Geisinger et al. This content is distributed under the terms of the Creative Commons Attribution 4.0 International license.

### Antibiotic susceptibility assays.

For growth curve analysis, cultures were seeded at an *A*_600_ of 0.003 in 100 μl of broth in wells of a 96-well microtiter plate and growth monitored during incubation at 37°C with orbital shaking in a Tecan M200 Pro plate reader. MIC tests were performed under the conditions described above using serial 2-fold dilutions of drug; the MIC was the lowest drug concentration preventing growth above *A*_600_ of 0.05 after 16 h. Colony-forming efficiency in the presence of ciprofloxacin was measured on LB agar plates containing graded concentrations of drug, as previously described (34). For determination of rifampin resistance frequency, bacteria were grown with or without ciprofloxacin for 3 h and pelleted by centrifugation. Dilutions of bacteria were spotted on LB agar to determine the total number of viable bacteria, and remaining bacteria were spread on LB agar plates with 50 μg/ml rifampin to determine rifampin-resistant mutant counts. After overnight incubation at 37°C, the frequency of rifampin mutants was calculated as the CFU per milliliter on LB-rifampin agar plates divided by the CFU per milliliter on LB agar plates.

### Construction of transposon mutant libraries.

Plasmid pDL1073 was employed for transposon mutagenesis. pDL1073 contains a kanamycin-resistant (Km^r^) Tn*10* derivative, an altered target-specificity Tn*10* transposase gene downstream of the phage lambda P_L_ promoter, a pSC101ts origin of replication, and β-lactamase (ampicillin resistant [Amp^r^]; Fig. S1A). pDL1073 does not replicate in A. baumannii at 37°C, allowing efficient detection at this temperature of transposition after delivery via electroporation. A. baumannii cells (50 μl) were combined with 100 ng pDL1073 and electroporated via a Bio-Rad Gene Pulser (0.1-cm-gap-length cuvette; 200Ω, 25 μF, and 1.8kV). Electroporated cells were diluted with super optimal broth with catabolite repression (SOC) broth and immediately spread onto membrane filters (0.45-μm pore size) overlaid on prewarmed LB agar plates. After incubating 2 h at 37°C, the filter membranes were transferred to prewarmed LB agar plates containing 20 μg/ml Km and incubated at 37°C overnight to select for transposon mutants. Bacterial colonies were lifted from the filter by agitation in sterile phosphate-buffered saline (PBS). Glycerol was added to 10% (vol/vol), and pooled mutant suspensions were aliquoted and stored at −80°C. Eleven to 15 independent pools each consisting of approximately 6,000 to 18,000 mutants were generated in each strain background.

### Tn-seq fitness measurements.

Transposon library aliquots were thawed, vortexed, diluted to an *A*_600_ of 0.1, and grown to an *A*_600_ of 0.2 in LB. Cultures were then back diluted to *A*_600_ of 0.003 in 10 ml LB without drug or with graded concentrations of ciprofloxacin. Parallel cultures were grown at 37°C for approximately 8 generations to an *A*_600_ of 0.5 to 1. Samples taken at the start (*t*_1_) and end (*t*_2_) of this outgrowth were stored at −20°C. Eleven to 15 independent transposon libraries were analyzed with each strain background. The use of multiple independent transposon mutant pools and high density of insertions across pools minimizes the contribution of adventitious secondary mutations to the determination of transposon mutant fitness. With WT libraries, treatments with 0.075 μg/ml and 0.09 to 0.1 μg/ml ciprofloxacin were performed in parallel with the same untreated control.

### Tn-seq Illumina library preparation.

Genomic DNA was extracted from *t*_1_ and *t*_2_ samples (Qiagen DNeasy kit) and quantified using a SYBR green microtiter assay. Transposon-adjacent DNA was amplified for Illumina sequencing using a modification of the Nextera DNA library prep method (Illumina). Thirty nanograms of genomic DNA was used as input in a 10-μl tagmentation reaction mixture. The reaction conditions were 55°C for 5 min, followed by inactivation at 95°C for 0.5min. Transposon-adjacent genomic DNA was amplified by adding 40 μl of PCR master mix containing primers olj928 and Nextera 2A-R (0.6 μM final) and Q5 high-fidelity polymerase (NEB). The reaction conditions were 98°C for 10 s, 65°C for 20 s, and 72°C for 1 min (30 cycles), followed by a final extension at 72°C for 2 min. A second PCR was performed using nested indexed primers. This reaction mixture contained 0.5 μl of the first PCR product, Left Tn*10* indexing primer (0.6 μM), Right indexing primer (0.6μM), and Q5 polymerase in a 50-μl final volume. The reaction conditions were 12 cycles of 98°C for 10 s, 65°C for 20 s, and 72°C for 1 min, followed by a final extension at 72˚C for 2 min. A sample of the second PCR product was imaged after separation on a 2% agarose–Tris-acetate-EDTA (TAE) gel containing SYBR Safe dye. Samples were multiplexed based on signal intensity in the bp 250 to 600 region and purified (Qiagen QIAquick kit). Fifteen to 20 pmol DNA was used as the template in a 50-μl reconditioning reaction mixture containing adapter-specific primers P1 and P2 (0.6 μM) and Q5 polymerase. The reaction conditions were 95°C for 1 min, 0.1°C/s ramp to 64°C, 64°C for 20 s, and 72°C for 10 min. Samples were purified (Qiagen QIAquick kit), followed by quantification and size selection (250 to 600 bp; Pippin HT) by the Tufts University Genomics Core Facility (TUCF-Genomics). Libraries were sequenced (single-end 50-bp reads) using custom primer olk115 on a HiSeq2500 with high-output V4 chemistry at TUCF-Genomics.

### Tn-seq data analysis.

Reads were demultiplexed, quality filtered, and clipped of adapters before serving as the input for mapping and fitness calculations (31). Reads were mapped to the A. baumannii 17978-mff chromosome (GenBank accession number NZ_CP012004) and plasmids (GenBank accession numbers CP000522.1, CP000523.1, and NZ_CP012005) using previously described parameters (61). Fitness values for each transposon mutant were calculated by comparing mutant versus population-wide expansion between *t*_1_ and *t*_2_ (31). Per-gene average fitness and standard deviation (SD) were then computed from fitness scores for all insertion mutations within a gene across multiple parallel transposon pools. Differences in average gene fitness between treated and untreated conditions (W_diff_) were considered significant if they fulfilled the following 3 criteria, with minor modifications from those previously described (33): per-gene fitness must be calculated from at least 3 data points, the magnitude of W_diff_ must be >10%, and the *q* value must be <0.05 in an unpaired *t* test, with FDR controlled by the 2-stage step-up method of Benjamini, Krieger, and Yekutieli (GraphPad Prism 7) (69). Per-insertion fitness scores within a given genomic region were visualized using the Integrative Genomics Viewer software (62) after aggregating all scores across multiple independent transposon mutant libraries using the SingleFitness Perl script (63).

### Whole-genome sequencing of individual strains subjected to ciprofloxacin.

WT, *gyrA*^r^ mutant, and *gyrA*^r^ *parC*^r^ mutant strains were grown from single colonies to early postexponential phase and back diluted to an *A*_600_ of 0.003. Parallel cultures were grown for 2.5 h in the absence of treatment or 3.5 h in the presence of ciprofloxacin treatment. DNA was extracted (Qiagen DNeasy kit), and Illumina sequencing libraries were amplified and sequenced as described previously (34). After mapping to NZ_CP012004, coverage files were generated from the resulting BAM files using deepTools, with reads normalized to counts per million (64).

### Transcriptional profiling.

Cultures were diluted to an *A*_600_ of 0.003 and grown for 2.5 h (untreated) or 3.5 h (ciprofloxacin treated). Cultures were mixed with an equal volume of ice-cold acetone-ethanol (1:1) and stored at –80°C. Cells were thawed and washed with Tris-EDTA (TE), and RNA was extracted (Qiagen RNeasy kit). RNA samples were diluted, combined with SUPERase-in (Invitrogen), and processed via the RNAtag-seq method (42). Illumina cDNA sequencing libraries were sequenced and reads processed as described previously (65). Differential expression was calculated using DESeq2 (66).

### Fluorescence reporter assays.

Strains containing pCC1 or pCC7 were cultured in the presence or absence of ciprofloxacin as in the RNA-seq experiments. Cells were immobilized on agarose pads and imaged on a Leica AF6000 microscope using a 100×/1.3 objective and TX2 filter cube (excitation, band pass [BP] 560/40; dichromatic mirror, 595; emission, BP 645/75). MicrobeJ (67) was used to measure the background-corrected mean fluorescence intensity per cell. Median cellular fluorescence intensities from populations of at least 100 bacteria were determined, and median values across multiple independent experiments were averaged.

### Data availability.

The sequencing reads analyzed in this study were deposited in the SRA database under accession number SRP157243 (Tn-seq) and BioProject numbers PRJNA495614 (RNA-seq) and PRJNA495623 (whole-genome sequencing).

## References

[1] Tacconelli E, Carrara E, Savoldi A, Harbarth S, Mendelson M, Monnet DL, Pulcini C, Kahlmeter G, Kluytmans J, Carmeli Y, Ouellette M, Outterson K, Patel J, Cavaleri M, Cox EM, Houchens CR, Grayson ML, Hansen P, Singh N, Theuretzbacher U, Magrini N, WHO Pathogens Priority List Working Group. 2018 Discovery, research, and development of new antibiotics: the WHO priority list of antibiotic-resistant bacteria and tuberculosis. Lancet Infect Dis 18:318–327. doi:10.1016/S1473-3099(17)30753-3. 29276051

[2] Warner WA, Kuang SN, Hernandez R, Chong MC, Ewing PJ, Fleischer J, Meng J, Chu S, Terashita D, English L, Chen W, Xu HH 2016 Molecular characterization and antimicrobial susceptibility of Acinetobacter baumannii isolates obtained from two hospital outbreaks in Los Angeles County, California, USA. BMC Infect Dis 16:194. doi:10.1186/s12879-016-1526-y. 27146090 PMC4857389

[3] Kim D, Ahn JY, Lee CH, Jang SJ, Lee H, Yong D, Jeong SH, Lee K 2017 Increasing resistance to extended-spectrum cephalosporins, fluoroquinolone, and carbapenem in Gram-negative bacilli and the emergence of carbapenem non-susceptibility in Klebsiella pneumoniae: analysis of Korean Antimicrobial Resistance Monitoring System (KARMS) data from 2013 to 2015. Ann Lab Med 37:231–239. doi:10.3343/alm.2017.37.3.231. 28224769 PMC5339095

[4] Blanco N, Harris AD, Rock C, Johnson JK, Pineles L, Bonomo RA, Srinivasan A, Pettigrew MM, Thom KA, the CDC Epicenters Program. 2018 Risk factors and outcomes associated with multidrug-resistant Acinetobacter baumannii upon intensive care unit admission. Antimicrob Agents Chemother 62:e01631-17. doi:10.1128/AAC.01631-17. PMC5740370 29133567

[5] Drlica K, Hiasa H, Kerns R, Malik M, Mustaev A, Zhao X 2009 Quinolones: action and resistance updated. Curr Top Med Chem 9:981–998. doi:10.2174/156802609789630947. 19747119 PMC3182077

[6] Hooper DC 1999 Mechanisms of fluoroquinolone resistance. Drug Resist Updat 2:38–55. doi:10.1054/drup.1998.0068. 11504468

[7] Jacoby GA 2005 Mechanisms of resistance to quinolones. Clin Infect Dis 41 Suppl 2:S120–S126. doi:10.1086/428052. 15942878

[8] Hujer KM, Hujer AM, Hulten EA, Bajaksouzian S, Adams JM, Donskey CJ, Ecker DJ, Massire C, Eshoo MW, Sampath R, Thomson JM, Rather PN, Craft DW, Fishbain JT, Ewell AJ, Jacobs MR, Paterson DL, Bonomo RA 2006 Analysis of antibiotic resistance genes in multidrug-resistant Acinetobacter sp. isolates from military and civilian patients treated at the Walter Reed Army Medical Center. Antimicrob Agents Chemother 50:4114–4123. doi:10.1128/AAC.00778-06. 17000742 PMC1694013

[9] Valentine SC, Contreras D, Tan S, Real LJ, Chu S, Xu HH 2008 Phenotypic and molecular characterization of Acinetobacter baumannii clinical isolates from nosocomial outbreaks in Los Angeles County, California. J Clin Microbiol 46:2499–2507. doi:10.1128/JCM.00367-08. 18524965 PMC2519477

[10] Fernando D, Zhanel G, Kumar A 2013 Antibiotic resistance and expression of resistance-nodulation-division pump- and outer membrane porin-encoding genes in Acinetobacter species isolated from Canadian hospitals. Can J Infect Dis Med Microbiol 24:17–21. doi:10.1155/2013/696043. 24421787 PMC3630023

[11] Rumbo C, Gato E, López M, Ruiz de Alegría C, Fernández-Cuenca F, Martínez-Martínez L, Vila J, Pachón J, Cisneros JM, Rodríguez-Baño J, Pascual A, Bou G, Tomás M, Spanish Group of Nosocomial Infections and Mechanisms of Action and Resistance to Antimicrobials (GEIH-GEMARA) from the Spanish Society of Clinical Microbiology and Infectious Diseases (SEIMC) and the Spanish Network for Research in Infectious Diseases (REIPI). 2013 Contribution of efflux pumps, porins, and beta-lactamases to multidrug resistance in clinical isolates of Acinetobacter baumannii. Antimicrob Agents Chemother 57:5247–5257. doi:10.1128/AAC.00730-13. 23939894 PMC3811325

[12] Yoon EJ, Chabane YN, Goussard S, Snesrud E, Courvalin P, De E, Grillot-Courvalin C 2015 Contribution of resistance-nodulation-cell division efflux systems to antibiotic resistance and biofilm formation in Acinetobacter baumannii. mBio 6:e00309-15. doi:10.1128/mBio.00309-15. 25805730 PMC4453527

[13] Poole K 2000 Efflux-mediated resistance to fluoroquinolones in Gram-negative bacteria. Antimicrob Agents Chemother 44:2233–2241. doi:10.1128/aac.44.9.2233-2241.2000. 10952561 PMC90051

[14] Recacha E, Machuca J, Diaz de Alba P, Ramos-Guelfo M, Docobo-Perez F, Rodriguez-Beltran J, Blazquez J, Pascual A, Rodriguez-Martinez JM 2017 Quinolone resistance reversion by targeting the SOS response. mBio 8:e00971-17. doi:10.1128/mBio.00971-17. 29018116 PMC5635686

[15] Cirz RT, Chin JK, Andes DR, de Crecy-Lagard V, Craig WA, Romesberg FE 2005 Inhibition of mutation and combating the evolution of antibiotic resistance. PLoS Biol 3:e176. doi:10.1371/journal.pbio.0030176. 15869329 PMC1088971

[16] Khodursky AB, Cozzarelli NR 1998 The mechanism of inhibition of topoisomerase IV by quinolone antibacterials. J Biol Chem 273:27668–27677. doi:10.1074/jbc.273.42.27668. 9765303

[17] Mo CY, Manning SA, Roggiani M, Culyba MJ, Samuels AN, Sniegowski PD, Goulian M, Kohli RM 2016 Systematically altering bacterial SOS Activity under stress reveals therapeutic strategies for potentiating antibiotics. mSphere 1:e00163-16. doi:10.1128/mSphere.00163-16. 27536734 PMC4980697

[18] Nichols RJ, Sen S, Choo YJ, Beltrao P, Zietek M, Chaba R, Lee S, Kazmierczak KM, Lee KJ, Wong A, Shales M, Lovett S, Winkler ME, Krogan NJ, Typas A, Gross CA 2011 Phenotypic landscape of a bacterial cell. Cell 144:143–156. doi:10.1016/j.cell.2010.11.052. 21185072 PMC3060659

[19] Sutherland JH, Tse-Dinh YC 2010 Analysis of RuvABC and RecG involvement in the Escherichia coli response to the covalent topoisomerase-DNA complex. J Bacteriol 192:4445–4451. doi:10.1128/JB.00350-10. 20601468 PMC2937393

[20] Tamae C, Liu A, Kim K, Sitz D, Hong J, Becket E, Bui A, Solaimani P, Tran KP, Yang H, Miller JH 2008 Determination of antibiotic hypersensitivity among 4,000 single-gene-knockout mutants of Escherichia coli. J Bacteriol 190:5981–5988. doi:10.1128/JB.01982-07. 18621901 PMC2519525

[21] Urios A, Herrera G, Aleixandre V, Blanco M 1990 Expression of the recA gene is reduced in Escherichia coli topoisomerase I mutants. Mutat Res 243:267–272. doi:10.1016/0165-7992(90)90142-7. 2157980

[22] Brazas MD, Breidenstein EB, Overhage J, Hancock RE 2007 Role of lon, an ATP-dependent protease homolog, in resistance of Pseudomonas aeruginosa to ciprofloxacin. Antimicrob Agents Chemother 51:4276–4283. doi:10.1128/AAC.00830-07. 17893152 PMC2167996

[23] Breidenstein EB, Khaira BK, Wiegand I, Overhage J, Hancock RE 2008 Complex ciprofloxacin resistome revealed by screening a Pseudomonas aeruginosa mutant library for altered susceptibility. Antimicrob Agents Chemother 52:4486–4491. doi:10.1128/AAC.00222-08. 18824609 PMC2592849

[24] van Opijnen T, Camilli A 2012 A fine scale phenotype-genotype virulence map of a bacterial pathogen. Genome Res 22:2541–2551. doi:10.1101/gr.137430.112. 22826510 PMC3514683

[25] Kelley WL 2006 Lex marks the spot: the virulent side of SOS and a closer look at the LexA regulon. Mol Microbiol 62:1228–1238. doi:10.1111/j.1365-2958.2006.05444.x. 17042786

[26] Robinson A, Brzoska AJ, Turner KM, Withers R, Harry EJ, Lewis PJ, Dixon NE 2010 Essential biological processes of an emerging pathogen: DNA replication, transcription, and cell division in Acinetobacter spp. Microbiol Mol Biol Rev 74:273–297. doi:10.1128/MMBR.00048-09. 20508250 PMC2884411

[27] MacGuire AE, Ching MC, Diamond BH, Kazakov A, Novichkov P, Godoy VG 2014 Activation of phenotypic subpopulations in response to ciprofloxacin treatment in Acinetobacter baumannii. Mol Microbiol 92:138–152. doi:10.1111/mmi.12541. 24612352 PMC4005408

[28] Aranda J, Bardina C, Beceiro A, Rumbo S, Cabral MP, Barbe J, Bou G 2011 Acinetobacter baumannii RecA protein in repair of DNA damage, antimicrobial resistance, general stress response, and virulence. J Bacteriol 193:3740–3747. doi:10.1128/JB.00389-11. 21642465 PMC3147500

[29] Saroj SD, Clemmer KM, Bonomo RA, Rather PN 2012 Novel mechanism for fluoroquinolone resistance in Acinetobacter baumannii. Antimicrob Agents Chemother 56:4955–4957. doi:10.1128/AAC.00739-12. 22733072 PMC3421888

[30] Gallagher LA, Lee SA, Manoil C 2017 Importance of core genome functions for an extreme antibiotic resistance trait. mBio 8:e01655-17. doi:10.1128/mBio.01655-17. 29233894 PMC5727411

[31] van Opijnen T, Camilli A 2013 Transposon insertion sequencing: a new tool for systems-level analysis of microorganisms. Nat Rev Microbiol 11:435–442. doi:10.1038/nrmicro3033. 23712350 PMC3842022

[32] van Opijnen T, Bodi KL, Camilli A 2009 Tn-seq: high-throughput parallel sequencing for fitness and genetic interaction studies in microorganisms. Nat Methods 6:767–772. doi:10.1038/nmeth.1377. 19767758 PMC2957483

[33] van Opijnen T, Dedrick S, Bento J 2016 Strain dependent genetic networks for antibiotic-sensitivity in a bacterial pathogen with a large pan-genome. PLoS Pathog 12:e1005869. doi:10.1371/journal.ppat.1005869. 27607357 PMC5015961

[34] Geisinger E, Mortman NJ, Vargas-Cuebas G, Tai AK, Isberg RR 2018 A global regulatory system links virulence and antibiotic resistance to envelope homeostasis in Acinetobacter baumannii. PLoS Pathog 14:e1007030. doi:10.1371/journal.ppat.1007030. 29795704 PMC5967708

[35] Drlica K, Zhao X 1997 DNA gyrase, topoisomerase IV, and the 4-quinolones. Microbiol Mol Biol Rev 61:377–392. 9293187 10.1128/mmbr.61.3.377-392.1997 PMC232616

[36] Su XZ, Chen J, Mizushima T, Kuroda T, Tsuchiya T 2005 AbeM, an H+-coupled Acinetobacter baumannii multidrug efflux pump belonging to the MATE family of transporters. Antimicrob Agents Chemother 49:4362–4364. doi:10.1128/AAC.49.10.4362-4364.2005. 16189122 PMC1251516

[37] Knauf GA, Cunningham AL, Kazi MI, Riddington IM, Crofts AA, Cattoir V, Trent MS, Davies BW 2018 Exploring the antimicrobial action of quaternary amines against Acinetobacter baumannii. mBio 9:e02394-17. doi:10.1128/mBio.02394-17. 29437928 PMC5801471

[38] Gomez JE, Kaufmann-Malaga BB, Wivagg CN, Kim PB, Silvis MR, Renedo N, Ioerger TR, Ahmad R, Livny J, Fishbein S, Sacchettini JC, Carr SA, Hung DT 2017 Ribosomal mutations promote the evolution of antibiotic resistance in a multidrug environment. Elife 6:e20420. doi:10.7554/eLife.20420. 28220755 PMC5319836

[39] Bollenbach T, Quan S, Chait R, Kishony R 2009 Nonoptimal microbial response to antibiotics underlies suppressive drug interactions. Cell 139:707–718. doi:10.1016/j.cell.2009.10.025. 19914165 PMC2838386

[40] Shippy DC, Fadl AA 2015 RNA modification enzymes encoded by the gid operon: Implications in biology and virulence of bacteria. Microb Pathog 89:100–107. doi:10.1016/j.micpath.2015.09.008. 26427881

[41] Hujer KM, Hujer AM, Endimiani A, Thomson JM, Adams MD, Goglin K, Rather PN, Pennella TT, Massire C, Eshoo MW, Sampath R, Blyn LB, Ecker DJ, Bonomo RA 2009 Rapid determination of quinolone resistance in Acinetobacter spp. J Clin Microbiol 47:1436–1442. doi:10.1128/JCM.02380-08. 19297590 PMC2681857

[42] Shishkin AA, Giannoukos G, Kucukural A, Ciulla D, Busby M, Surka C, Chen J, Bhattacharyya RP, Rudy RF, Patel MM, Novod N, Hung DT, Gnirke A, Garber M, Guttman M, Livny J 2015 Simultaneous generation of many RNA-seq libraries in a single reaction. Nat Methods 12:323–325. doi:10.1038/nmeth.3313. 25730492 PMC4712044

[43] Simmons LA, Foti JJ, Cohen SE, Walker GC 2008 The SOS regulatory network. EcoSal Plus 3. doi:10.1128/ecosalplus.5.4.3. 26443738

[44] Hare JM, Ferrell JC, Witkowski TA, Grice AN 2014 Prophage induction and differential RecA and UmuDAb transcriptome regulation in the DNA damage responses of Acinetobacter baumannii and Acinetobacter baylyi. PLoS One 9:e93861. doi:10.1371/journal.pone.0093861. 24709747 PMC3978071

[45] Ching C, Gozzi K, Heinemann B, Chai Y, Godoy VG 2017 RNA-mediated cis regulation in Acinetobacter baumannii modulates stress-induced phenotypic variation. J Bacteriol 199:e00799-16. doi:10.1128/JB.00799-16. 28320880 PMC5424255

[46] Chao MC, Abel S, Davis BM, Waldor MK 2016 The design and analysis of transposon insertion sequencing experiments. Nat Rev Microbiol 14:119–128. doi:10.1038/nrmicro.2015.7. 26775926 PMC5099075

[47] Erb ML, Kraemer JA, Coker JK, Chaikeeratisak V, Nonejuie P, Agard DA, Pogliano J 2014 A bacteriophage tubulin harnesses dynamic instability to center DNA in infected cells. Elife 3. doi:10.7554/eLife.03197. PMC4244570 25429514

[48] Constantinou A, Voelkel-Meiman K, Sternglanz R, McCorquodale MM, McCorquodale DJ 1986 Involvement of host DNA gyrase in growth of bacteriophage T5. J Virol 57:875–882. 2419589 10.1128/jvi.57.3.875-882.1986 PMC252817

[49] Alonso JC, Sarachu AN, Grau O 1981 DNA gyrase inhibitors block development of Bacillus subtilis bacteriophage SP01. J Virol 39:855–860. 6270354 10.1128/jvi.39.3.855-860.1981 PMC171318

[50] Alcorlo M, Salas M, Hermoso JM 2007 In vivo DNA binding of bacteriophage GA-1 protein p6. J Bacteriol 189:8024–8033. doi:10.1128/JB.01047-07. 17873040 PMC2168694

[51] Kreuzer KN, Cozzarelli NR 1979 Escherichia coli mutants thermosensitive for deoxyribonucleic acid gyrase subunit A: effects on deoxyribonucleic acid replication, transcription, and bacteriophage growth. J Bacteriol 140:424–435. 227840 10.1128/jb.140.2.424-435.1979 PMC216666

[52] Itoh T, Tomizawa JI 1977 Involvement of DNA gyrase in bacteriophage T7 DNA replication. Nature 270:78–80. doi:10.1038/270078a0. 927524

[53] Sokolsky TD, Baker TA 2003 DNA gyrase requirements distinguish the alternate pathways of Mu transposition. Mol Microbiol 47:397–409. doi:10.1046/j.1365-2958.2003.03296.x. 12519191

[54] Dodson M, Echols H, Wickner S, Alfano C, Mensa-Wilmot K, Gomes B, LeBowitz J, Roberts JD, McMacken R 1986 Specialized nucleoprotein structures at the origin of replication of bacteriophage lambda: localized unwinding of duplex DNA by a six-protein reaction. Proc Natl Acad Sci U S A 83:7638–7642. doi:10.1073/pnas.83.20.7638. 3020552 PMC386776

[55] Mensa-Wilmot K, Seaby R, Alfano C, Wold MC, Gomes B, McMacken R 1989 Reconstitution of a nine-protein system that initiates bacteriophage lambda DNA replication. J Biol Chem 264:2853–2861. 2536726

[56] Kreuzer KN 1998 Bacteriophage T4, a model system for understanding the mechanism of type II topoisomerase inhibitors. Biochim Biophys Acta 1400:339–347. doi:10.1016/S0167-4781(98)00145-6. 9748648

[57] Huang WM, Wei LS, Casjens S 1985 Relationship between bacteriophage T4 and T6 DNA topoisomerases. T6 39-protein subunit is equivalent to the combined T4 39- and 60-protein subunits. J Biol Chem 260:8973–8977. 2991231

[58] Sutton MD, Smith BT, Godoy VG, Walker GC 2000 The SOS response: recent insights into umuDC-dependent mutagenesis and DNA damage tolerance. Annu Rev Genet 34:479–497. doi:10.1146/annurev.genet.34.1.479. 11092836

[59] Di Nocera PP, Rocco F, Giannouli M, Triassi M, Zarrilli R 2011 Genome organization of epidemic Acinetobacter baumannii strains. BMC Microbiol 11:224. doi:10.1186/1471-2180-11-224. 21985032 PMC3224125

[60] Geisinger E, Isberg RR 2015 Antibiotic modulation of capsular exopolysaccharide and virulence in Acinetobacter baumannii. PLoS Pathog 11:e1004691. doi:10.1371/journal.ppat.1004691. 25679516 PMC4334535

[61] Carter R, Wolf J, van Opijnen T, Muller M, Obert C, Burnham C, Mann B, Li Y, Hayden RT, Pestina T, Persons D, Camilli A, Flynn PM, Tuomanen EI, Rosch JW 2014 Genomic analyses of pneumococci from children with sickle cell disease expose host-specific bacterial adaptations and deficits in current interventions. Cell Host Microbe 15:587–599. doi:10.1016/j.chom.2014.04.005. 24832453 PMC4066559

[62] Robinson JT, Thorvaldsdottir H, Winckler W, Guttman M, Lander ES, Getz G, Mesirov JP 2011 Integrative genomics viewer. Nat Biotechnol 29:24–26. doi:10.1038/nbt.1754. 21221095 PMC3346182

[63] McCoy KM, Antonio ML, van Opijnen T 2017 MAGenTA: a Galaxy implemented tool for complete Tn-Seq analysis and data visualization. Bioinformatics 33:2781–2783. doi:10.1093/bioinformatics/btx320. 28498899 PMC5860071

[64] Ramírez F, Dundar F, Diehl S, Gruning BA, Manke T 2014 deepTools: a flexible platform for exploring deep-sequencing data. Nucleic Acids Res 42:W187–W191. doi:10.1093/nar/gku365. 24799436 PMC4086134

[65] Jensen PA, Zhu Z, van Opijnen T 2017 Antibiotics disrupt coordination between transcriptional and phenotypic stress responses in pathogenic bacteria. Cell Rep 20:1705–1716. doi:10.1016/j.celrep.2017.07.062. 28813680 PMC5584877

[66] Love MI, Huber W, Anders S 2014 Moderated estimation of fold change and dispersion for RNA-seq data with DESeq2. Genome Biol 15:550. doi:10.1186/s13059-014-0550-8. 25516281 PMC4302049

[67] Ducret A, Quardokus EM, Brun YV 2016 MicrobeJ, a tool for high throughput bacterial cell detection and quantitative analysis. Nat Microbiol 1:16077. doi:10.1038/nmicrobiol.2016.77. 27572972 PMC5010025

[68] Arndt D, Grant JR, Marcu A, Sajed T, Pon A, Liang Y, Wishart DS 2016 PHASTER: a better, faster version of the PHAST phage search tool. Nucleic Acids Res 44:W16–W21. doi:10.1093/nar/gkw387. 27141966 PMC4987931

[69] Benjamini Y, Krieger AM, Yekutieli D 2006 Adaptive linear step-up procedures that control the false discovery rate. Biometrika 93:491–507. doi:10.1093/biomet/93.3.491.

