# Supplementary figures and images for: The Landscape of Phenotypic and Transcriptional Responses to Ciprofloxacin in Acinetobacter baumannii: Acquired Resistance Alleles Modulate Drug-Induced SOS Response and Prophage Replication
Source: mBio. 2019 Jun 11;10(3):e01127-19. doi: 10.1128/mBio.01127-19 (PMC6561030; doi:10.1128/mBio.01127-19)

**A**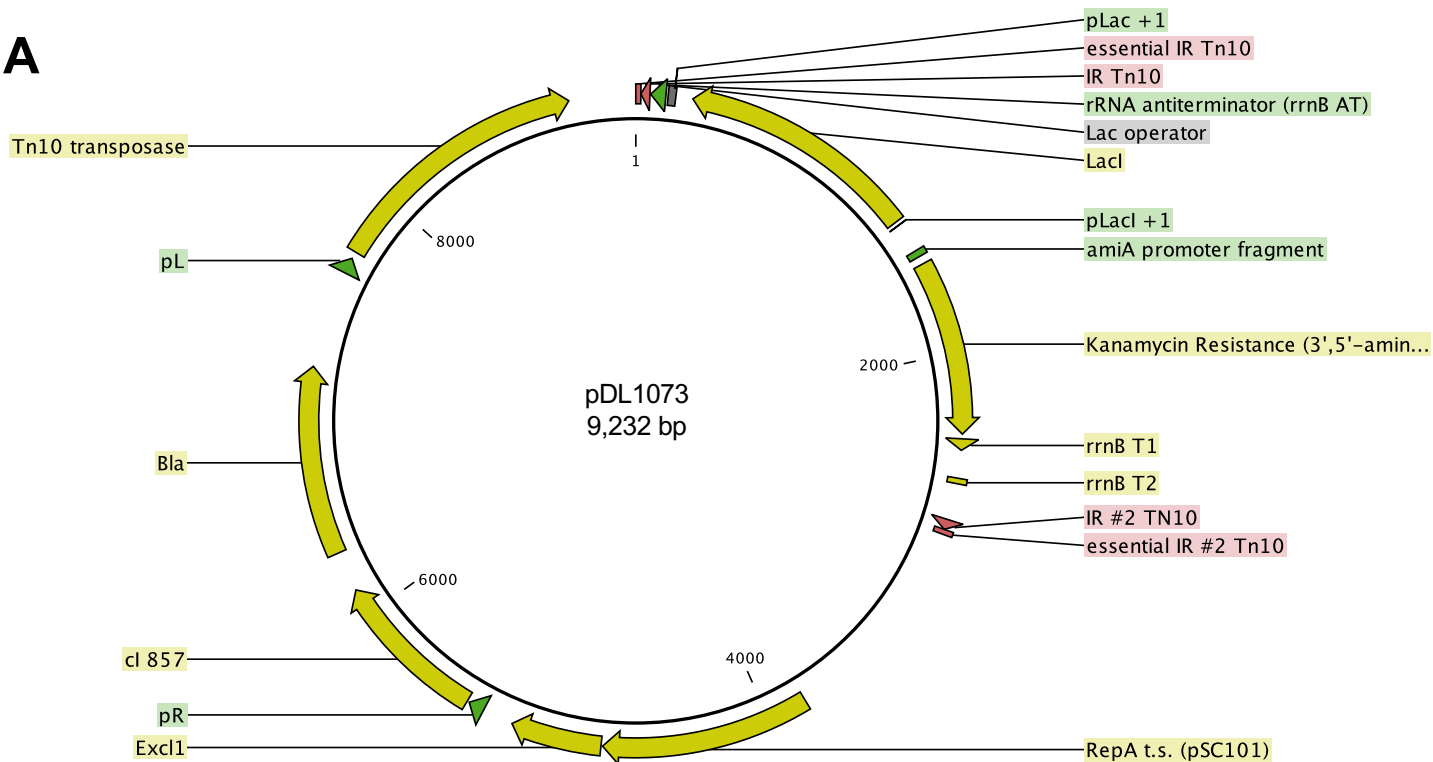**B**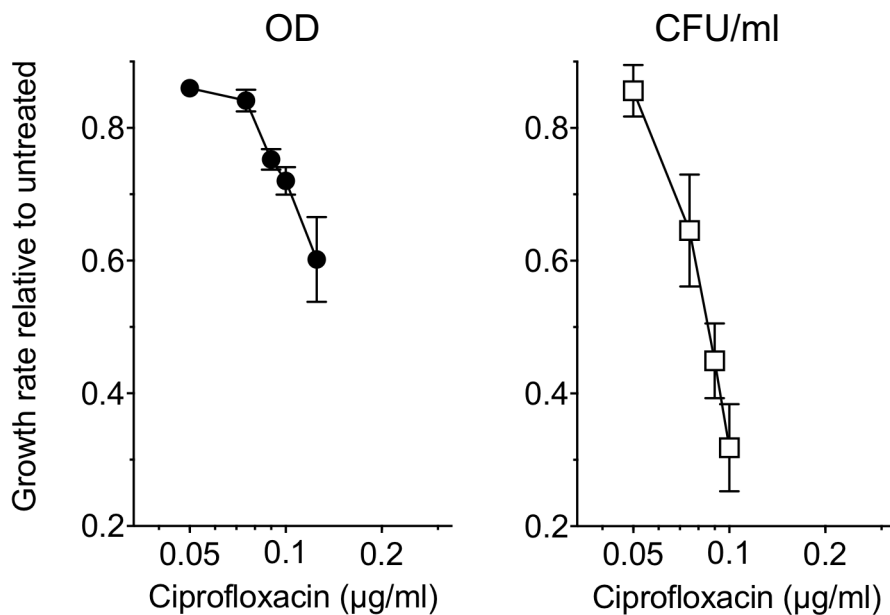

Supplement: FIG S1 [file mBio.01127-19-sf001.pdf]

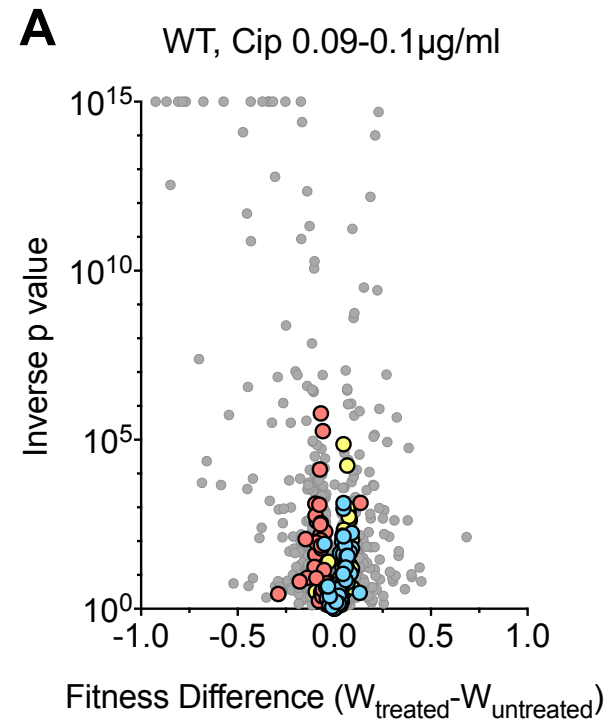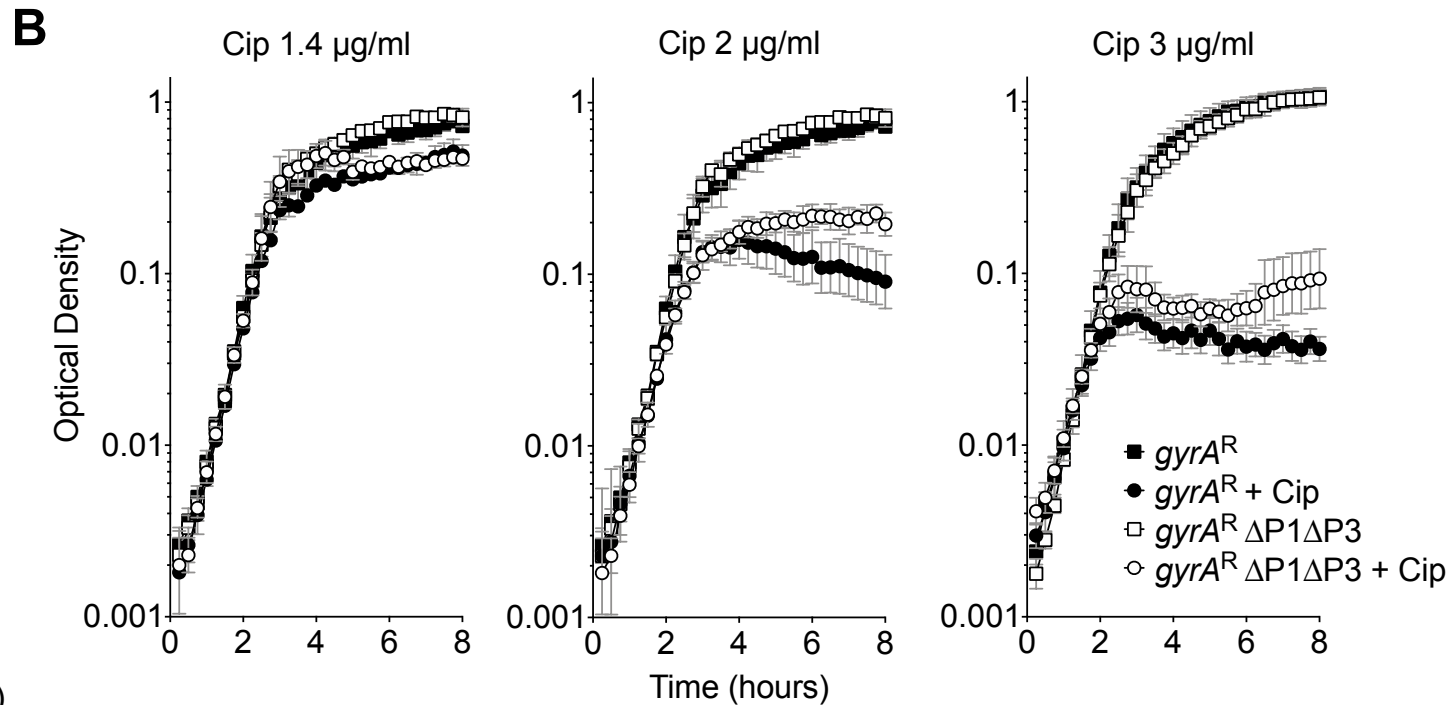

Supplement: FIG S2 [file mBio.01127-19-sf002.pdf]

■ WT  
■ *gyrA*(S81L)

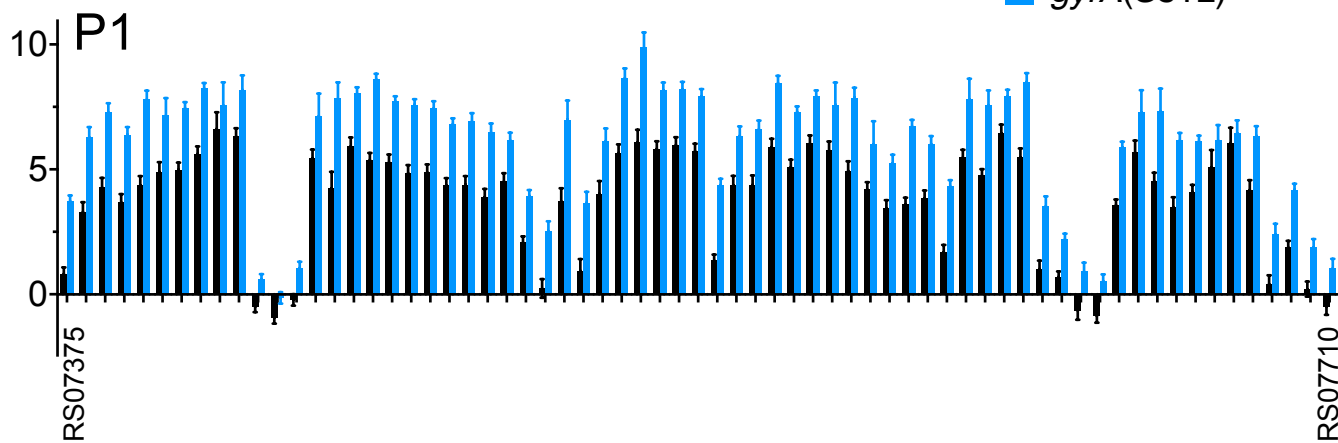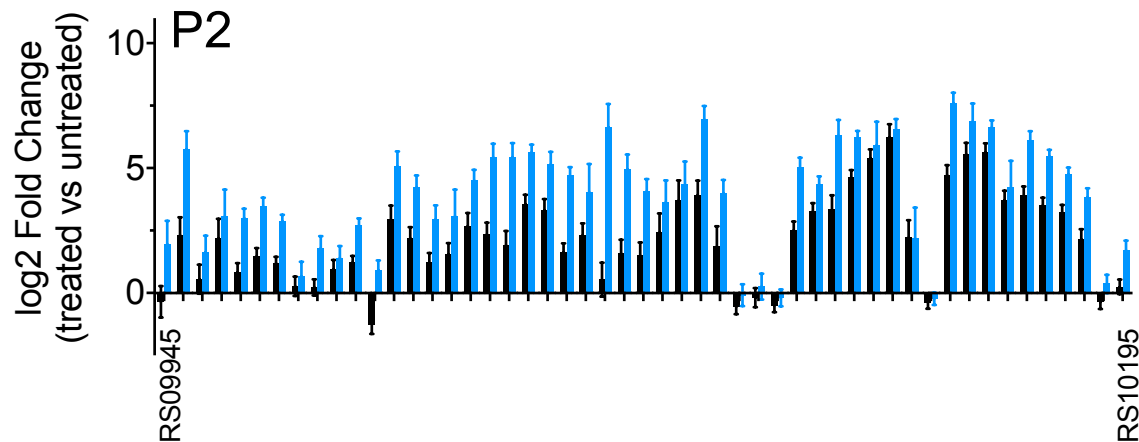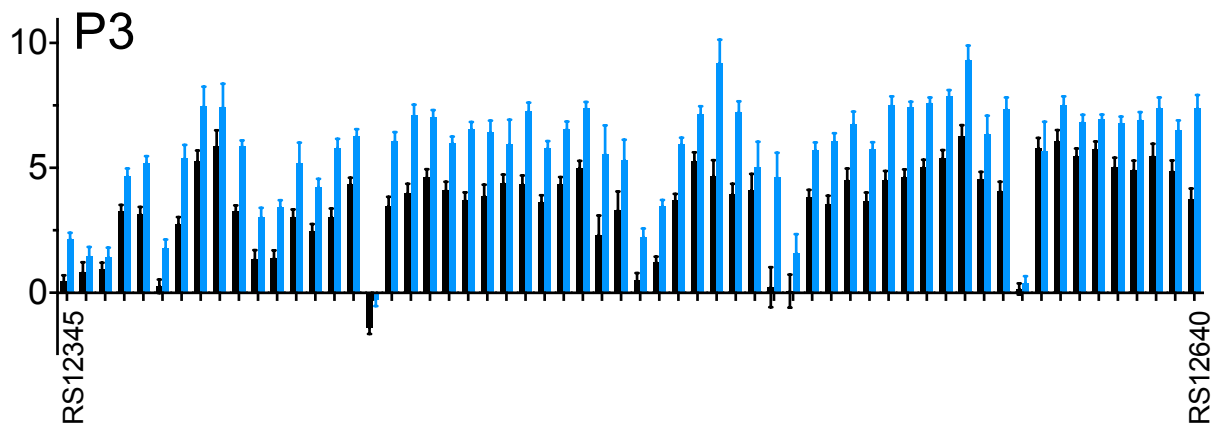

Supplement: FIG S3 [file mBio.01127-19-sf003.pdf]

WT

*gyrA<sup>R</sup>**gyrA<sup>R</sup>parC<sup>R</sup>*

untreated

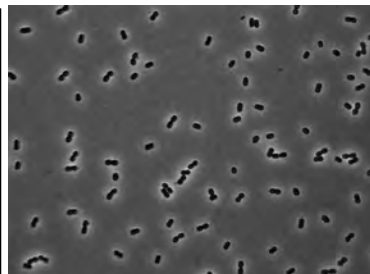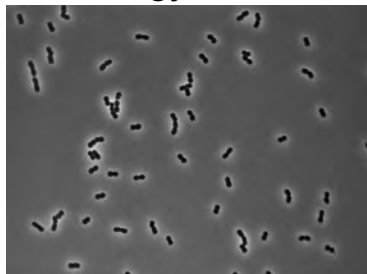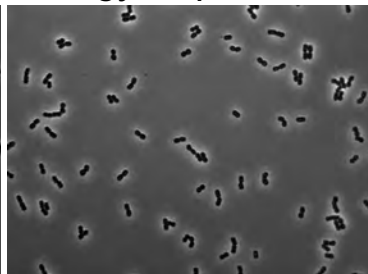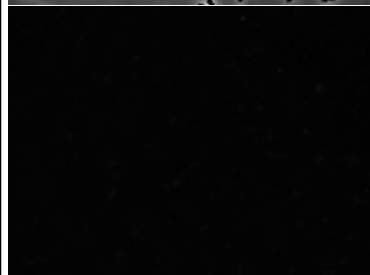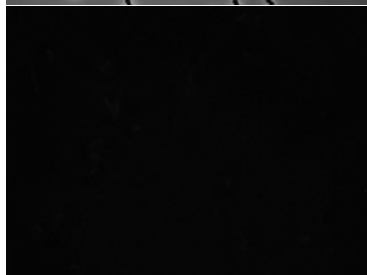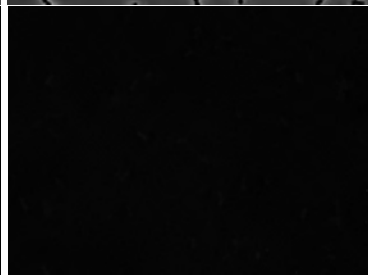

+ ciprofloxacin

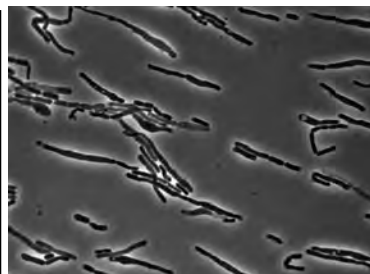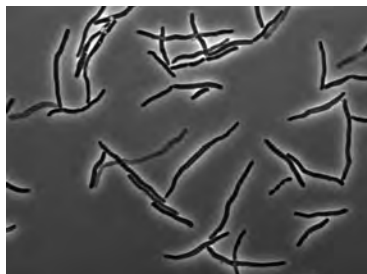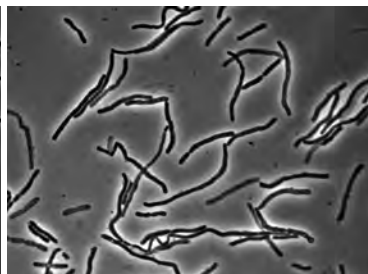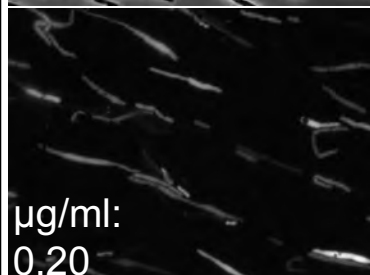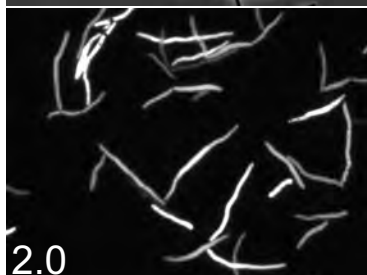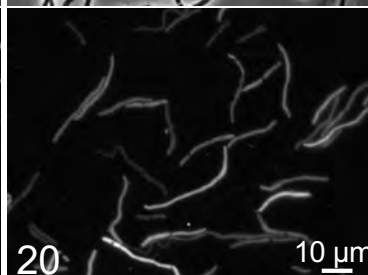

Supplement: FIG S4 [file mBio.01127-19-sf004.pdf]
