# Supplementary material for: The Landscape of Phenotypic and Transcriptional Responses to Ciprofloxacin in Acinetobacter baumannii: Acquired Resistance Alleles Modulate Drug-Induced SOS Response and Prophage Replication
Source: mBio. 2019 Jun 11;10(3):e01127-19. doi: 10.1128/mBio.01127-19 (PMC6561030; doi:10.1128/mBio.01127-19)
Supplement: TABLE S1 [file mBio.01127-19-st001.pdf]

S1 Table. Strains and plasmids.

Strain or plasmid	Genotype or description	Reference
A. baumannii		
ATCC 17978	cerebrospinal fluid isolate	(1)
EGA336	ATCC 17978 *gyrA*(S81L)	This study
GVA41	ATCC 17978 *parC*(S84L)	This study
GVA37	ATCC 17978 *gyrA*(S81L) *parC*(S84L)	This study
EGA465	ATCC 17978 Δ*adeIJK*	(2)
GVA68	ATCC 17978 Δ*recN*	This study
GVA84	ATCC 17978 Δ*ctpA*	This study
EGA627	ATCC 17978 *pbp1a*(N178TfsX27)	This study
GVA61	ATCC 17978 Δ*gidA*	This study
YDA124	ATCC 17978 ΔP1 ΔP3	This study
E. coli		
DH5α	*supE44* Δ*lacU169* (ϕ80lacZΔM15) *hsdR17 recA1 endA1 gyrA96 thi*-1 *relA1*	(3)
DH5λpir	DH5α (λpir) *tet*::Mu *recA*	(4)
TO60	DH5α λpir [F′ *proAB lacI*^{q}ZΔM15 Tn10 (Tc^R)]	(5)
**plasmids**		
pUC18	*oriColE1* MCS Cb^R	(6)
pSR47s	*oriTRP4 oriR6K* Km^R	(7)
pDL1073	Tn10 (Km^R) delivery plasmid, *ori pSC101* Cb^R	This study
pWH1266	shuttle plasmid, Cb^R Tet^R	(8)
pEGE244	pWH1266 with *rrnB* & T7 tandem terminators replacing EcoRI-PstI fragment, Tet^R	(2)
pGVE41	pWH1266 with *ctpA* replacing EcoRI-PstI fragment, Tet^R	This study
pCC1	*recA* promoter-UTR(C::G)-mKate2 transcriptional fusion in pNLAC1 vector, Tet^R	(9)
pCC7	*trpB* promoter-UTR(C::G)-mKate2 transcriptional fusion in pNLAC1 vector, Tet^R	(9)

References

1. Bouvet P, Grimont P. 1986. Taxonomy of the Genus *Acinetobacter* with the Recognition of *Acinetobacter baumannii* sp. nov. *Acinetobacter haemolyticus* sp. nov. *Acinetobacter johnsonii* sp. nov. and *Acinetobacter junii* sp. nov. and Emended Descriptions of *Acinetobacter calcoaceticus* and *Acinetobacter lwofii*. International Journal of Systematic Bacteriology 36:228-240.
2. Geisinger E, Mortman NJ, Vargas-Cuebas G, Tai AK, Isberg RR. 2018. A global regulatory system links virulence and antibiotic resistance to envelope homeostasis in *Acinetobacter baumannii*. PLoS Pathog 14:e1007030.
3. Hanahan D, Jessee J, Bloom FR. 1991. Plasmid transformation of *Escherichia coli* and other bacteria. Methods Enzymol 204:63-113.
4. Kolter R, Inuzuka M, Helinski DR. 1978. Trans-complementation-dependent replication of a low molecular weight origin fragment from plasmid R6K. Cell 15:1199-208.

5. O'Connor TJ, Adepoju Y, Boyd D, Isberg RR. 2011. Minimization of the *Legionella pneumophila* genome reveals chromosomal regions involved in host range expansion. Proc Natl Acad Sci U S A 108:14733-40.
6. Yanisch-Perron C, Vieira J, Messing J. 1985. Improved M13 phage cloning vectors and host strains: nucleotide sequences of the M13mp18 and pUC19 vectors. Gene 33:103-19.
7. Andrews HL, Vogel JP, Isberg RR. 1998. Identification of linked *Legionella pneumophila* genes essential for intracellular growth and evasion of the endocytic pathway. Infect Immun 66:950-8.
8. Hunger M, Schmucker R, Kishan V, Hillen W. 1990. Analysis and nucleotide sequence of an origin of DNA replication in *Acinetobacter calcoaceticus* and its use for *Escherichia coli* shuttle plasmids. Gene 87:45-51.
9. Ching C, Gozzi K, Heinemann B, Chai Y, Godoy VG. 2017. RNA-Mediated cis Regulation in *Acinetobacter baumannii* Modulates Stress-Induced Phenotypic Variation. J Bacteriol 199.
