# Supplementary material for: The Landscape of Phenotypic and Transcriptional Responses to Ciprofloxacin in Acinetobacter baumannii: Acquired Resistance Alleles Modulate Drug-Induced SOS Response and Prophage Replication
Source: mBio. 2019 Jun 11;10(3):e01127-19. doi: 10.1128/mBio.01127-19 (PMC6561030; doi:10.1128/mBio.01127-19)
Supplement: TABLE S2 [file mBio.01127-19-st002.pdf]

S2 Table. Oligonucleotide primers

**Cloning**		
**primer name**	**Sequence (5' – 3'; restriction site underlined, with partial sites in italics)**	**RE site(s)**
gyrA_Lbam	CTGTGGATCCAGTTGGCGAAGTAAAAGATGC	BamHI
gyrA_Rsal	TTTGGTCGACAGTTAATACCACTTGCTCTTCAGG	SalI
gyrA-S81L-R	AGTCACCATGCGGGTGATATTTAC	
gyrA-S81L-F	TAGCTGTTTATGAAACCATTGTTCG	
parC_F4 NotI	CCTGGCGGCCGCACGCTGGTCAGTGAATATGTGC	NotI
parC_R5 SalI	TATTGTCGACGCCAGACGCTAGCTTTATCTCTG	SalI
parC NcoI 3	GAGTACCATGGCTTCATAACATGCCAAGTCACCATGTGGGTGGTATTTA	NcoI
recN-up-F	GACCGCGGCCGCATCAAATTGGGTTGAACTCATGC	NotI
recN-up-R	TTAAGAGCTCGAGCATAAACGCATCCAACG	SacI
recN-down-F	TTCTGAGCTCTAATAAAGAAAATGAGAGAAAAAGATTGG	SacI
recN-down-R	GCATGTCGACATCGTAAGCAGCTTTATGTTGTGG	SalI
ctpA_up-F	ATATGAGCTCTTGCAGCATTTAGTCACCTATCG	SacI
ctpA_up-R	GCAAGCGGCCGCAATTTAACTGCAATGAAAGCAAAGC	NotI
ctpA_down-F	TAAAGTCGACTTTAGTACCGAAATTAATGGAACTGG	SalI
ctpA_down-R	TCTAGAGCTCAAGTAAATTTTATACTTCCTGTTCTGTCTCC	SacI
gidA-up-R	CATCGAGCTCTTTAGGATAGTGCATGAGTACAAAAACACC	SacI
gidA-up-F	TCTTGCGGCCGCCCAGCAAGTGCGAAATCACC	NotI
gidA-down-R	AAGCGTCGACAAACCGAATCTGACAAACTTGC	SalI
gidA-down-F	GAAAGAGCTCGCTTAATTATTTAAGCGTGTAAAAAAGC	SacI
ctpA-comp-F	AACTGAATTCAGATTCCTGTGCCTGCTGAAATGC	EcoRI
ctpA-comp-R	CTAACTGCAGAGGAATGACTTTCCGCTCTTTACGC	PstI
**Tn-seq sequencing library construction**		
**primer name**	**Sequence (5' – 3')**	**index**
First PCR		
Nextera 2A-R	GTCTCGTGGGCTCGGAGATGTGTATAAGAGACAG	
olj928	CTGTGTGGGCACTCGACATATGAC	
Second PCR -- Leftward TN10 specific Nextera Indexed primers		
Olk147	AATGATACGGCGACCACCGAGATCTACACGCAGGCGGCCGGGGGCCAAAATCATTAGGGGATTCATCAG	GCAGGCGG
Olk148	AATGATACGGCGACCACCGAGATCTACACAGGCAGAACCGGGGGCCAAAATCATTAGGGGATTCATCAG	AGGCAGAA
Olk149	AATGATACGGCGACCACCGAGATCTACACCAGAGAGGCCGGGGGCCAAAATCATTAGGGGATTCATCAG	CAGAGAGG
Olk150	AATGATACGGCGACCACCGAGATCTACACCGAGGCTGCCGGGGGCCAAAATCATTAGGGGATTCATCAG	CGAGGCTG
Olk151	AATGATACGGCGACCACCGAGATCTACACAAGAGGCACCGGGGGCCAAAATCATTAGGGGATTCATCAG	AAGAGGCA
Olk152	AATGATACGGCGACCACCGAGATCTACACGAGGAGCCCCGGGGGCCAAAATCATTAGGGGATTCATCAG	GAGGAGCC
Olk153	AATGATACGGCGACCACCGAGATCTACACAGCGCAGACCGGGGGCCAAAATCATTAGGGGATTCATCAG	AGCGCAGA
Olk110	AATGATACGGCGACCACCGAGATCTACACGTAAGGAGCCGGGGGCCAAAATCATTAGGGGATTCATCAG	GTAAGGAG
Olk154	AATGATACGGCGACCACCGAGATCTACACAACCCGGACCGGGGGCCAAAATCATTAGGGGATTCATCAG	AACCCGGA
Olk155	AATGATACGGCGACCACCGAGATCTACACGCGGAAGCCCGGGGGCCAAAATCATTAGGGGATTCATCAG	GCGGAAGC
Second PCR -- Rightward Nextera Indexed primers		
olk141	CAAGCAGAAGACGGCATACGAGATCCGCCTGCGTCTCGTGGGCTCGGAGATGTG	GCAGGCGG

N703 index	CAAGCAGAAGACGGCATACGAGATTTCTGCCTGTCTCGTGGGCTCGGAGATGTG	AGGCAGAA
N708 index	CAAGCAGAAGACGGCATACGAGATCCTCTCTGGTCTCGTGGGCTCGGAGATGTG	CAGAGAGG
N710 index	CAAGCAGAAGACGGCATACGAGATCAGCCTCGGTCTCGTGGGCTCGGAGATGTG	CGAGGCTG
N711 index	CAAGCAGAAGACGGCATACGAGATTGCCTCTTGTCTCGTGGGCTCGGAGATGTG	AAGAGGCA
olk142	CAAGCAGAAGACGGCATACGAGATGGCTCCTCGTCTCGTGGGCTCGGAGATGTG	GAGGAGCC
olk143	CAAGCAGAAGACGGCATACGAGATTCTGCGCTGTCTCGTGGGCTCGGAGATGTG	AGCGCAGA
olk144	CAAGCAGAAGACGGCATACGAGATCTCCTTACGTCTCGTGGGCTCGGAGATGTG	GTAAGGAG
olk145	CAAGCAGAAGACGGCATACGAGATTCCGGGTTGTCTCGTGGGCTCGGAGATGTG	AACCCGGA
olk146	CAAGCAGAAGACGGCATACGAGATGCTTCCGCGTCTCGTGGGCTCGGAGATGTG	GCGGAAGC
Reconditioning		
P1	AATGATACGGCGACCACCGA	
P2	CAAGCAGAAGACGGCATACGA	
Sequencing		
olk115	CCGGGGGCCAAAATCATTAGGGGATTCATCAG	
